# The ethyl acetate extract from *Trichoderma viride* fermentation acts by downregulating the leukocyte transendothelial migration signaling pathway to induce ferroptosis in triple-negative breast cancer cells

**DOI:** 10.1007/s13659-025-00569-w

**Published:** 2026-01-10

**Authors:** Yu Kuang, Bai-Hui Lu, Jia-Yi Wu, Song-Yu Wu, Hai-Yan Fu, Qing-Yan Nan, Jing Li, Xiao-Long Yang

**Affiliations:** 1 School of Pharmaceutical Sciences, South-Central Minzu University, Wuhan, 430074 China; 2 School of Pharmacy, Anhui University of Chinese Medicine, Hefei, 230012 China; 3 College of Life Sciences, South-Central Minzu University, Wuhan, 430074 China

**Keywords:** *Trichoderma viride*, Triple-negative breast cancer, Leukocyte transendothelial migration, Ferroptosis

## Abstract

**Purpose:**

Triple-negative breast cancer (TNBC), characterized by the absence of estrogen receptor (ER), progesterone receptor (PR), and human epidermal growth factor receptor 2 (HER2) expression, remains clinically challenging due to the lack of effective targeted therapies. This investigation revealed the anti-TNBC potential of *Trichoderma viride* ethyl acetate extract (TVEAE) from the endophytic fungus *Trichoderma viride* isolated from *Coreopsis basalis*.

**Methods:**

Pharmacological validation of TVEAE's anti-TNBC efficacy was conducted through in vitro and in vivo pharmacological models. The cell death mechanisms were systematically investigated using Hoechst staining, reactive oxygen species (ROS) detection, and lipid peroxidation assays. Potential therapeutic targets and signaling pathways were identified by integrating network pharmacology, transcriptomics, and weighted gene co-expression network analysis (WGCNA). Furthermore, this study validated key tumor-related proteins involved in tumor progression and cell death pathways via Western blotting. Finally, chemical constituents were characterized through molecular network coupled with Global Natural Products Social Molecular Networking (GNPS) analysis.

**Results:**

Both in vitro and in vivo models established TVEAE's significant anti-TNBC efficacy. Mechanistic interrogation established TVEAE-mediated ferroptosis induction via selective modulation of leukocyte transendothelial migration (TEM) signaling cascades. Integrative analysis combining transcriptomics, WGCNA, and network pharmacology identified IL-6/TNF-*α*/HSP90AA1 as core therapeutic targets regulating TEM pathway dynamics. GNPS-assisted molecular networking uncovered six structurally novel anti-TNBC metabolites, including N-lauryldiethanolamine, erucamide, and Gliotoxin.

**Conclusion:**

This study provides the first evidence of TVEAE's anti-TNBC activity through multi-target engagement along the leukocyte TEM signaling axis, effectively triggering ferroptosis. The mechanistic elucidation advances TNBC therapeutic development, offering a multi-dimensional targeting strategy against this recalcitrant malignancy.

**Supplementary Information:**

The online version contains supplementary material available at 10.1007/s13659-025-00569-w.

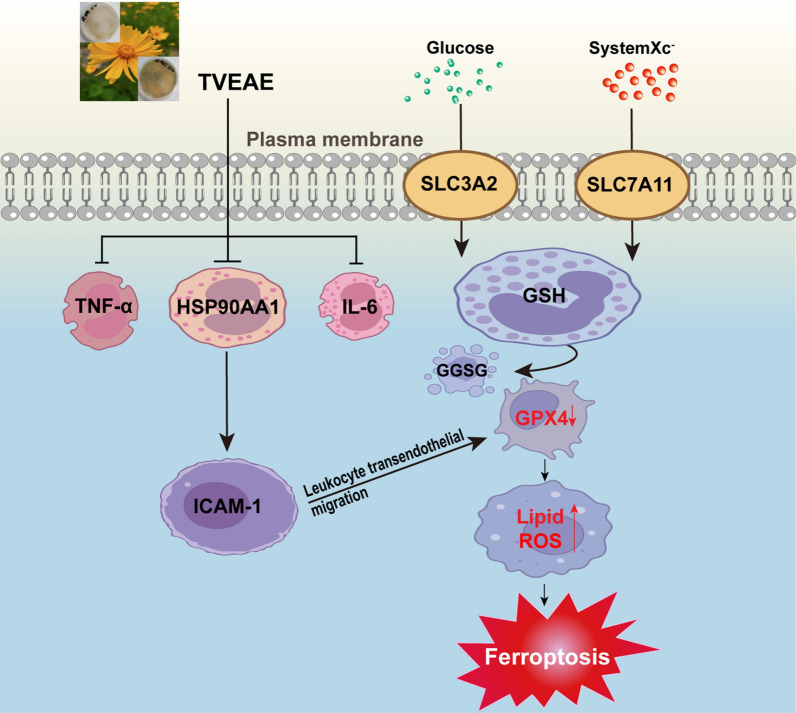

## Introduction

Triple-negative breast cancer (TNBC), characterized by the absence of estrogen receptor (ER), progesterone receptor (PR), and human epidermal growth factor receptor 2 (HER2) expression, is a highly aggressive subtype unresponsive to endocrine and targeted therapies [1–4]. Accounting for 15–20% of breast cancer (BC) cases globally, TNBC is associated with a significantly lower five-year survival rate due to its rapid progression and high metastatic potential [5–8]. As the most common malignant tumor among women worldwide, BC poses a serious public health threat. Epidemiological data show that 1 out of every 20 women worldwide is diagnosed with this disease [9–11]. In China, the disease burden of BC is rising at a rapid pace: between 2000 and 2015, its incidence rate increased annually by 3.3%, and by 2022, this rate had reached 45.29 cases per 100,000 population [12, 13]. Although the overall mortality rate has declined slightly, delayed diagnosis remains a critical issue. Evidence shows that the early detection rate is below 20%, and cases detected through screening account for less than 5% of the total diagnosed cases, which is particularly true among high-risk groups such as women aged 65–69 [14]. The current treatment strategies for TNBC remain suboptimal. Conventional chemotherapy and immunotherapy have limited efficacy, with a five-year survival rate of less than 80% [15]. These challenges underscore the urgent need for innovative drug development to establish safer, more effective precision treatment paradigms for this recalcitrant malignancy [16–19].

Natural products demonstrate unparalleled value in oncotherapeutic development through their polypharmacological mechanisms and superior toxicological tolerability [20–22]. Notably, approximately 60% of clinically approved anticancer agents originate directly from natural products or their structurally optimized derivatives, escalating to 80% when considering natural product-inspired synthetic analogs [23, 24]. Endophytic fungi establish a mutualistic symbiotic relationship with host plants. They can not only promote the synthesis of plant secondary metabolites but also secrete bioactive defensive compounds [25, 26]. Recent studies have revealed that the metabolic synergy between host plants and endophytic fungi can produce new antitumor substances [27]. For instance, the chemical screening of endophytic fungus *Aspergillus japonicus* TE-739D isolated from tobacco obtaining the compound aspergillamide B (ATB). This compound induces MFC cell apoptosis in a concentration-dependent manner and promotes the generation of reactive oxygen species (ROS) in MFC cells. In addition, ATB significantly inhibited tumor growth in a subcutaneously transplanted tumor model derived from MFC cells [28]. This “plant–microbe” metabolic network synergy establishes a new discovery pipeline for bioactive lead compounds.

Plants of the *Coreopsis* genus, as traditional medicinal resources, have recently been found to possess unique anticancer potential. For instance, the anti-tumor activity of *Coreopsis* against lung cancer may be related to its inhibition of the PI3K-Akt signaling pathway and activation of the mitochondrial-mediated apoptosis pathway [29]. Based on existing theoretical research, we selected the endophytic fungi from *Coreopsis basalis* as the research subject, aiming to screen functional strains with TNBC activity. Notably, we discovered that the ethyl acetate extract from *Trichoderma virens* significantly inhibit the growth of MDA-MB-231 cells, indicating potent anti-TNBC activity.

Ferroptosis is an iron-dependent form of programmed cell death that has been discovered in recent years. It is characterized by abnormal iron accumulation and lipid peroxidation [30, 31]. Its core mechanism involves the following: ferroptosis-inducing factors regulate glutathione peroxidase activity through multiple pathways, leading to impairment of the intracellular antioxidant system, accumulation of ROS, and ultimately oxidative stress-mediated cell death [32]. Deng Rong's research group at Sun Yat-sen University first proposed a ferroptosis induction strategy targeting lysosomal exocytosis, providing a new therapeutic direction for Akt-driven refractory tumors [33]. This fully indicates that ferroptosis is of great significance in inducing tumor cell death. In this study, we validated the antitumor activity of the fermentation product against TNBC through in vitro and in vivo experiments. Ferrous ion content assays suggested that its anti-TNBC mechanism is associated with the induction of ferroptosis. Furthermore, transcriptome sequencing and subsequent Gene Ontology (GO) and Kyoto Encyclopedia of Genes and Genomes (KEGG) enrichment analyses predicted potential pathways and targets, providing critical clues for further investigation. This data was analyzed by combining in vitro experiments, in vivo models, and transcriptome sequencing. The results revealed that TVEAE inhibited TNBC by inducing ferroptosis. Specifically, TVEAE modulates key nodes in the leukocyte TEM signaling pathway, upregulates intercellular adhesion molecule-1 (ICAM-1) expression to trigger sustained ROS accumulation, and significantly downregulates glutathione peroxidase 4 (GPX4) expression, collectively triggering ferroptotic cell death.

## Materials and methods

### Materials

This strain was isolated from the medicinal plant *Coreopsis basalis* collected in Pingjiang County, Hunan Province. The internal transcribed spacer (ITS) region of the rDNA was amplified. and molecular identification confirmed the strain as *Trichoderma viride* (GenBank accession no. PX530447). The culture has been deposited at South-Central Minzu University.

Ethyl acetate was purchased from Sinopharm Chemical Reagent Co., Ltd. Cell Counting Kit-8 (CCK8) kit was purchased from ABclonal Biotechnology Co., Ltd. The ferrous ion detection kit was purchased from Ruixin Biotechnology Co., Ltd. Hoechst 33,342 and crystal violet were sourced from Beyotime (C1062S, Beyotime Biotechnology Inc, Shanghai, China). The microscope was purchased from Guangzhou Haokang Biotechnology Co., Ltd. LC–MS grade methanol, formic acid, and acetonitrile were purchased from Thermo Fisher Scientific (Fairlawn, NJ, USA). Ultrapure water was purified using a Milli-Q Integral Water Purification System (Millipore, USA).

### Fermentation and extraction

The fungal strain *Trichoderma viride* was inoculated onto potato dextrose agar (PDA) medium (procured from AoBoXing Bio-Tech Co., Ltd, Wuhan, China) and cultured at 28 ℃ for 5 days to initiate sporulation. The culture was transferred to sterilized rice medium (100 g rice + 80 mL distilled water) and maintained under static incubation at 28 ℃ for 35 days. The fermented material was subjected to triple ethanol extraction, and the combined supernatants were concentrated *in vacuo* to yield a crude ethanolic extract. This crude extract was homogenized in distilled H_2_O, followed by extract with equivalent volumes of ethyl acetate. The ethyl acetate fractions were separated, combined, and concentrated *in vacuo* to yield the target extract.

### Cell culture and cell viability assay

The MDA-MB-231 cells (procured from Warner Bio, Wuhan, China) were cultured in Dulbecco’s modified Eagle medium, high glucose (Biosharp, Beijing, China), supplemented with 10% (v/v) fetal bovine serum (FBS) (Meilun Bio, Dalian, China) and 1% penicillin–streptomycin (P/S) (Tianhang, Shanghai, China). The MDA-MB-231 cells were subjected to medium replacement every day and cultured in a CO_2_ incubator with a concentration of 5% at 37 ℃.

TVEAE was dissolved in dimethyl sulfoxide (PanReac AppliChem, Beijing, China) to prepare a stock solution of 30 mM and diluted with culture medium before use to achieve the desired concentration. The MDA-MB-231 cells were seeded at a density of 1 × 10^5^ cells per well in 96-well culture plates. The cells were subsequently exposed to different concentrations of TVEAE for a duration of 24 h. After administration, cell viability was evaluated using the CCK8 assay. The activity of TVEAE was quantified as the cell inhibition rate and compared to that of cisplatin.

### Wound healing assay

The effects of the active compounds on cancer cell invasion and migration were preliminarily evaluated using the wound healing assay. For the wound healing assay, uniform horizontal lines were drawn on the back of a 6-well plate at 0.5 cm intervals prior to cell seeding. MDA-MB-231 cells were treated with varying concentrations of the compounds and cultured until a confluent monolayer was formed. A sterile pipette tip was used to create a scratch perpendicular to the pre-drawn lines, and the dislodged cells were removed by washing with phosphate buffered saline (PBS). The cells were then incubated in a 5% CO_2_ incubator for 24 h to allow migration. After crystal violet staining, images were captured using an inverted fluorescence microscope.

### Establishment of a BALB/c-nu nude mouse xenograft tumor model using 4T1 cells

All study experiments adhered to the National Institutes of Health Guidelines on the Use of Laboratory Animals and were approved by the South-Central Minzu University Committee on Animal Care. All experimental animals, female BALB/c-nu nude mice, were purchased from Jiangsu Huachuang-Xinnuo Pharmaceutical Technology Co., LTD.

In this study, log-phase 4T1 cells were adjusted to a concentration of 3 × 10^7^ cells/mL in culture medium. A 200 µL aliquot of the cell suspension was inoculated subcutaneously into the right axilla of 6-week-old female BALB/c-nu nude mice. The mice were then randomly allocated into five groups (5 mice per group): Model group (untreated tumor-bearing mice), Positive control group (standard drug treatment), Low-dose group (50 mg/kg), High-dose group (100 mg/kg), and Normal control group (non-tumor-bearing mice without 4T1 cell inoculation). Tumor growth was monitored by measuring subcutaneous tumor diameters every two days using calipers. Tumor volume was calculated using the formula V = (a^2^ × b) × 0.5, where a represents the short diameter and b the long diameter. Drug administration was initiated when the tumor volume in any mouse reached 150–200 mm^3^.

### Hematoxylin and eosin staining

The tumor tissues and organ samples (including liver and kidney) were processed as follows: First, the tissues were embedded in paraffin and sectioned into 4-μm-thick slices, followed by baking and dewaxing. After gradient ethanol hydration, the sections were stained with hematoxylin for 3 min. Then, they were rinsed under running water for 3 min. Next, the sections underwent differentiation, water washing, and bluing treatment. After that, they were rinsed under running water for another 10 min. Subsequently, eosin staining was carried out. The sections were then dehydrated using a graded ethanol series, mounted, and finally observed under a microscope (200 ×) for image acquisition.

### RNA extraction, cDNA library preparation, and illumina sequencing

First, total animal Ribonucleic Acid (RNA) was extracted using the TRIzol Kit (Life Technologies, Inc., California, USA). Subsequently, the concentration and purity of RNA were measured using a NanoDrop 2000 (Thermo Fisher Scientific, Wilmington, DE). The integrity of RNA was evaluated with the RNA Nano 6000 Assay Kit on the Agilent Bioanalyzer 2100 system (Agilent Technologies, CA, USA). The total amount of each sample used for library construction initiation was 1 μg. The sequencing libraries were generated using the Hieff NGS Ultima Dual-mode messenger RNA (mRNA) Library Prep Kit for Illumina (Yeasen Biotechnology Co., Ltd.), and indexes were added to the sequences of each sample. mRNA was retrieved from total RNA using magnetic beads with oligo. First-strand complementary DNA (cDNA) was synthesized, followed by the synthesis of second-strand cDNA. The protruding end at the tail is repaired into a blunt end through the activities of exonuclease and polymerase. Purify the library fragments using AMPure XP magnetic beads (Beckman Coulter, Beverly, USA). Then, 3 μL of USER Enzyme (NEB, USA) was added, followed by incubation at 37 ℃ for 15 min and a reaction at 95 ℃ for 5 min before Polymerase Chain Reaction (PCR).

The libraries were sequenced on the Illumina NovaSeq platform, generating 150 bp paired-end sequences. Raw data in Fastq format is first processed using an in-house Perl script. In this step, clean data is obtained by removing adapter-containing sequences, poly-N sequences, and low-quality sequences from the raw data. Meanwhile, Q20, Q30, GC content, and sequence duplication levels are calculated. All downstream analyses are based on high-quality clean data. The clean data were aligned to the reference genome sequences. Based on the reference genome, only sequences with perfect matches or one mismatch were subjected to further analysis and annotation. The Hisat2 software tool was used for alignment with the reference genome. Gene functions were annotated through sequence alignment based on the following databases: Nr (NCBI non-redundant protein sequences); Pfam (Protein family); KOG/COG (Clusters of Orthologous Groups of proteins); KO (KEGG Ortholog database).

### Ferrous iron content determination

Tissue-Level Analysis: Approximately 0.1 g of tumor tissue was homogenized in 1 mL of extraction buffer using an ice bath. The homogenate was centrifuged at 12,000 rpm for 5 min at 4 ℃, and the supernatant was collected and kept on ice for subsequent analysis. The iron ion detection reagent was added to the supernatant, thoroughly mixed, and incubated at room temperature for 15 min. Subsequently, 200 µL of the mixture was transferred to a 96-well plate, and the absorbance was measured at a wavelength of 562 nm. The ferrous iron content was calculated using the standard formula.

Cellular-Level Analysis: Cells (5 × 10^6^) were resuspended in 1 mL of ice-cold extraction buffer and lysed by ultrasonication (200 W output, 30 cycles of 3-s pulses with 10-s intervals, maintained on ice). The lysate was centrifuged at 12,000*g* for 10 min at 4 ℃ to obtain the supernatant. For iron quantification, the supernatant was mixed with iron detection reagent, incubated at room temperature for 15 min, and 200 µL aliquots were transferred to a 96-well plate. Absorbance was measured at 562 nm using a microplate reader, and ferrous iron concentration was calculated based on the standard curve.

### ROS staining experiment

Place the glass slide in PBS and wash it three times on a decolorization shaker, each time for 5 min. After air-drying, add 4',6-diamidino-2-phenylindole (DAPI) staining solution to the circle, followed by incubation at room temperature in the dark for 10 min. Next, wash the slide again in PBS on the decolorization shaker (three time, 5 min each), air-dry slightly and seal it with anti-fluorescence quenching mounting mediun. Finally, observe the slide and capture images under a fluorescence microscope.

### Lipid peroxidation staining experiment

Cells were pelleted by centrifugation and the supernatant was discarded. The cell pellet was resuspended in extraction buffer (1:500, v/v) and subjected to ultrasonication (ice bath, 20% or 200 W, 3-s pulses at 10-s intervals, 30 cycles). The lysate was centrifuged at 8,000 × g for 10 min at 4 ℃, and the supernatant was collected and kept on ice foe analysis. Preheat the spectrophotometer for at least 30 min, adjust the wavelength to 535 nm, and zero the distilled water. Add 900 μL of reagent 1, 300 μL of reagent 2, and 90 μL of sample in sequence into the Eppendorf (EP) tube. Add 900 μL of reagent 1,300 μL of reagent 2, and 90 μL of distilled water to the blank tube. After immediate vortexing, the mixture was incubated for 40 min, rapidly cooled in tap water following heating at 95 ℃ (if applicable), and centrifuged at 3000 × *g* for 10 min. The clarified supernatant (1 mL) was transferred to a 1-mL glass cuvette and absorbance was measured at 535 nm. The net absorbance (Δ A) was calculated as A measurement—A blank.

### LC–MS analysis of TVEAE

LC–MS analysis was performed on a UHPLC Q Exactive Orbitrap mass spectrometry system (ThermoFisher, USA) equipped with a Waters Acquity UHPLC HSS T3 column (2.1 × 100 mm, 1.8 µm). Ionization was achieved via an electrospray ionization (ESI) source operated in dual-polarity switching mode under the following optimized parameters: full scan mode (*m/z* 100–1500); ESI source voltages were 3.5 kV (positive ionization mode) and 2.5 kV (negative ionization mode); capillary temperature 320 °C, and source temperature 300 °C; sheath gas flow rate 35 arb, and auxiliary gas flow rate 10 arb.

Chromatographic separation was achieved at a flow rate of 0.2 mL/min with the column temperature maintained at ambient conditions. The injection volume was 5 µL, and detection wavelengths were set at 210 nm and 254 nm. The mobile phase consisted of solvent A (0.1% acetic acid in water) and solvent B (acetonitrile). A gradient elution program was employed as follows: 0–40 min, 5%–100% B; 40–50 min, 100% B.

### Identification of compounds in TVEAE using molecular networking

The LC–MS/MS raw data were converted to mzXML format using ProteoWizard (https://proteowizard.sourceforge.io/). MZmine 2 (v2.53) was employed for peak detection, alignment, and MS/MS spectrum extraction. A feature table containing precursor ion *m/z*, retention time, and fragment ion information was generated, and exported in.mgf format for subsequent analysis.

Molecular networking was conducted on the GNPS platform (https://gnps.ucsd.edu) with the following parameters: precursor ion mass tolerance (± 0.02 Da), fragment ion mass tolerance (± 0.02 Da), minimum cosine similarity threshold (0.7), and minimum number of matched fragments (6). We enabled the spectrum clustering and dereplication functions to optimize the network topology.

The data results were compared against the built-in mass spectrometry library of GNPS, and high-confidence annotations with a cosine similarity > 0.7 and a matched number of fragment ions ≥ 6 were retained. For unmatched nodes, we performed putative annotation of analogs by inferring structural modifications based on their co-clustering relationships with annotated nodes in the molecular network, combined with molecular weight differences. The results were visualized using Cytoscape (v3.7.1), with nodes colored according to bioactivity metadata to identify target compounds.

### Identification of compounds based on LC–MS/MS and spectral library matching

Compound identification was performed using the Thermo Scientific™ Xcalibur™ software package (Version 2.2). Raw mass spectrometry data were initially analyzed via the Qual Browser component, where extracted ion chromatograms (XICs) were generated for target ions using a 5 ppm mass accuracy window. MS/MS spectra were acquired at chromatographic peak apexes with dynamic exclusion to minimize redundant fragmentation. Experimental data were systematically compared against a custom spectral library derived from the PubChem database (https://pubchem.ncbi.nlm.nih.gov/) containing accurate parent ion *m/z* values, retention times (RT), and secondary fragment spectral.

### Identification of TVEAE therapeutic targets and venn diagram analysis

The compounds identified from TVEAE were analyzed using the PubChem database to obtain their Canonical SMILES identifiers. These SMILES identifiers were subsequently queried in the Swiss TargetPrediction database (http://swisstargetprediction.ch/) to predict potential compound targets. After removing duplicate entries, the corresponding official human gene symbols for these targets were retrieved using the UniProt database (https://www.uniprot.org/).

To identify TNBC-related targets, the GeneCards database (https://www.genecards.org/) was employed. The predicted compound targets and TNBC-associated targets were then analyzed using Venny 2.1.0 (https://bioinfogp.cnb.csic.es/tools/venny/index.html) to generate a Venn diagram, enabling the identification of overlapping targets. These overlapping targets represent the potential anti-TNBC targets of the compounds derived from TVEAE.

### Weighted correlation network analysis

The weighted gene co-expression network analysis (WGCNA) algorithm was implemented using the “WGCNA” R package (v1.72) to investigate the genetic mechanisms underlying tumor pathogenesis. Genes exhibiting the top 25% variance in the genome-wide transcriptomic dataset were selected for co-expression network construction. A scale-free topology criterion (soft thresholding power *β* = 7, fit index R^2^ = 0.80) was applied to optimize network connectivity. Dynamic tree-cutting with a height threshold of 0.25 was performed to merge highly correlated modules. Additional parameters included a minimum module size of 30 genes and correlation analysis between module eigengenes and clinical traits. GO and KEGG enrichment analyses were subsequently applied to functionally characterize key modules.

### Transcriptomic data analysis

The gene expression matrix generated from upstream transcriptomic analysis was subjected to differential expression analysis using Differential Expression analysis for Sequence data 2 (DESeq2) on the “Wei Sheng Xin” platform (https://www.bioinformatics.com.cn/). Subsequently, the processed data were analyzed for GO and KEGG pathway enrichment. The analyses were conducted with “Mus musculus” set as the species parameter. The GO enrichment analysis encompassed three categories: Biological Process (BP), Cellular Component (CC), and Molecular Function (MF). Additionally, KEGG pathway analysis was performed. Significant enrichment was observed in GO terms with a *p*-value threshold of 0.05, while KEGG pathway analysis revealed enriched pathways with a more stringent *p*-value threshold of 0.001. The results were visualized to elucidate the biological processes and signaling pathways associated with the therapeutic effects of TVEAE in TNBC.

### Western blot

Firstly, extract the sample protein and determine its concentration using the Bradford method. Add the sample buffer and boil for denaturation. Subsequently, Sodium Dodecyl Sulfate–Polyacrylamide Gel Electrophoresis (SDS-PAGE) was performed to separate proteins by molecular weight, and after completion, the proteins were transferred to the polyvinylidene difluoride (PVDF) membrane. Then block with Bovine Serum Albumin (BSA) at room temperature for 1 h to prevent non-specific binding. Add primary antibody and incubate overnight at 4 ℃. After washing the membrane with Tris-Buffered Saline with Tween (TBST), add Horseradish Peroxidase-labeled secondary antibody and incubate at room temperature for 1 h. Finally, develop with Enhanced Chemiluminescence (ECL) chemiluminescence reagent, capture the signal through an imaging system, and perform grayscale analysis. Attention should be paid to several key steps. First, thoroughly wash with TBST between each step. Second, dilute the primary and secondary antibodies according to the instructions. Third, activate the PVDF membrane with methanol for 30 s before transferring the membrane.

### Molecular docking

The 3D chemical structures of the TVEAE compounds were retrieved from the PubChem database. The core 3D structures targets were obtained from the Research Collaboratory for Structural Bioinformatics Protein Data Bank (RCSB PDB) (https://www.rcsb.org/). The 3D structures of compounds were uploaded to Discovery Studio (2019) for pre-processing of the small molecule. The proteins with the best target resolution were selected, namely HSP90AA1, TNF-*α* and IL-6. Subsequently, these proteins underwent a series of preparatory treatments upon importation into Discovery Studio (2019). After the compounds and proteins were prepared, LibDock docking was performed to investigate the interaction between the compounds and each protein. The binding energies of these interactions were subsequently calculated.

### Statistical analysis

The data were analyzed using Microsoft Excel. The experimental results were subjected to statistical analysis using the statistical software GraphPad Prism 8 (GraphPad Software, USA). Student’s t-test and one-way ANOVA were used to evaluate differences between groups. A value of *p* < 0.05 was considered to be statistically significant. ns, no significance; *p < 0.05; **p < 0.01; ***p < 0.001; ****p < 0.0001. #*p* < 0.05; ##*p* < 0.01; ###*p* < 0.001; ####*p* < 0.0001.

## Results

### TVEAE inhibited the proliferation of MDA-MB-231 cells in vitro

**Fig.1:**
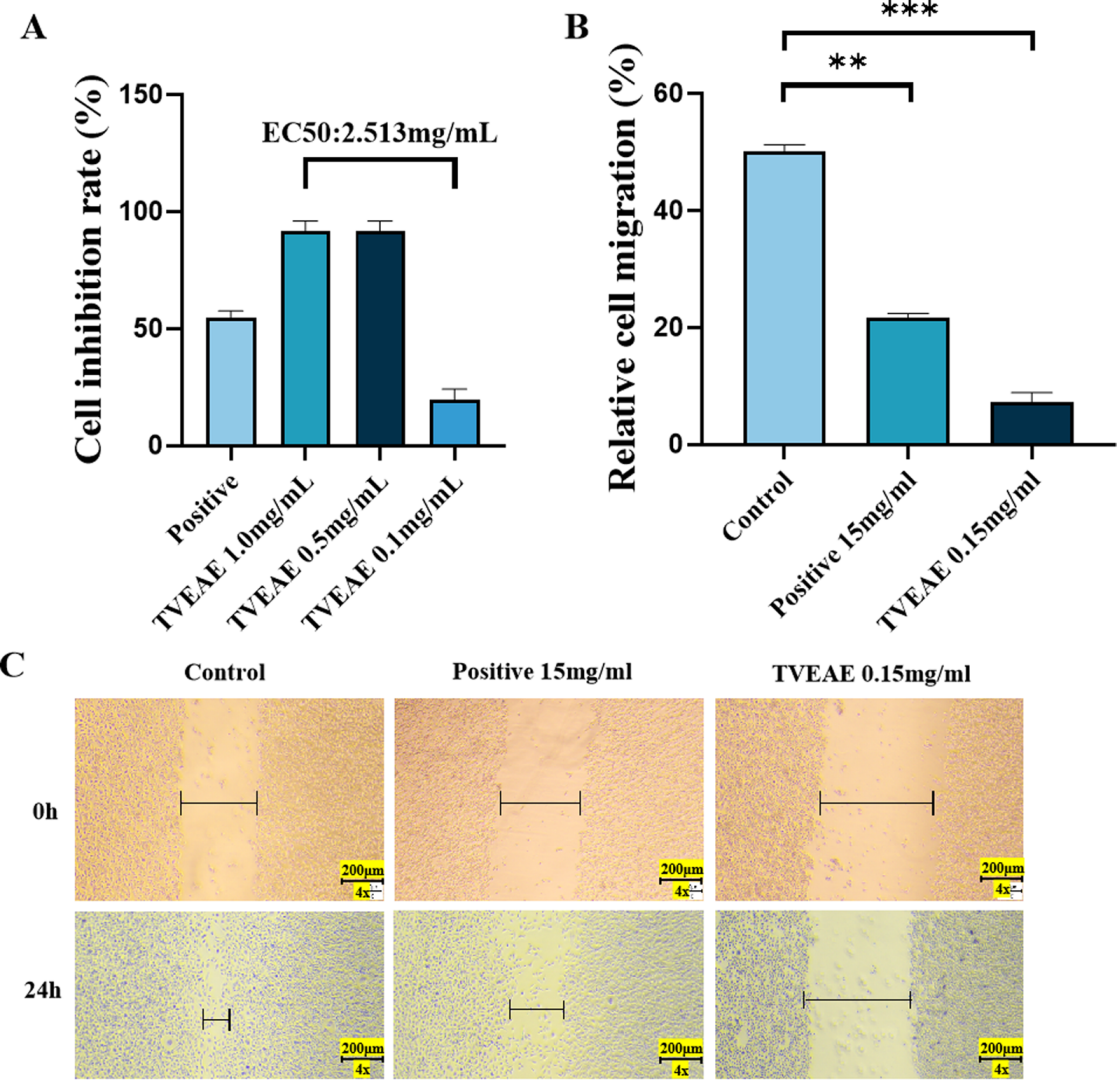
The CCK8 assay evaluated the cytostatic rate of TVEAE on MDA-MB-231 cells (**A**). Microscopy images of the wound healing assay at different times and quantification of relative cell migration in wound healing assays (**B**, **C**). The results are expressed as mean ± SD, *p < 0.05, **p < 0.01, ***p < 0.001, ****p < 0.0001 (n = 3 per group, where n represents three technical replicates in a single experiment)

CCK8 assay results demonstrated that TVEAE exhibited a concentration-dependent inhibitory effect on the viability of MDA-MB-231 cells. Compared with the positive control group, TVEAE at concentrations of 0.5 and 1.0 mg/mL significantly inhibited cell proliferation (*p* < 0.05). The median effect concentration (EC50) of TVEAE was 2.513 mg/mL (Fig. 1A). Tumor cell migration is a hallmark of tumor progression and metastasis. To evaluate the effect of TVEAE on cell migration, we adopted the classic wound healing assay. The results showed that compared with the control group, where the wound size was significantly reduced, there was no obvious change in wound closure in the TVEAE administered group (Fig. 1B, C). Overall, TVEAE inhibited the proliferation and migration of MDA-MB-231 cells in vitro.

### TVEAE significantly suppressed tumor growth in vivo

**Fig.2:**
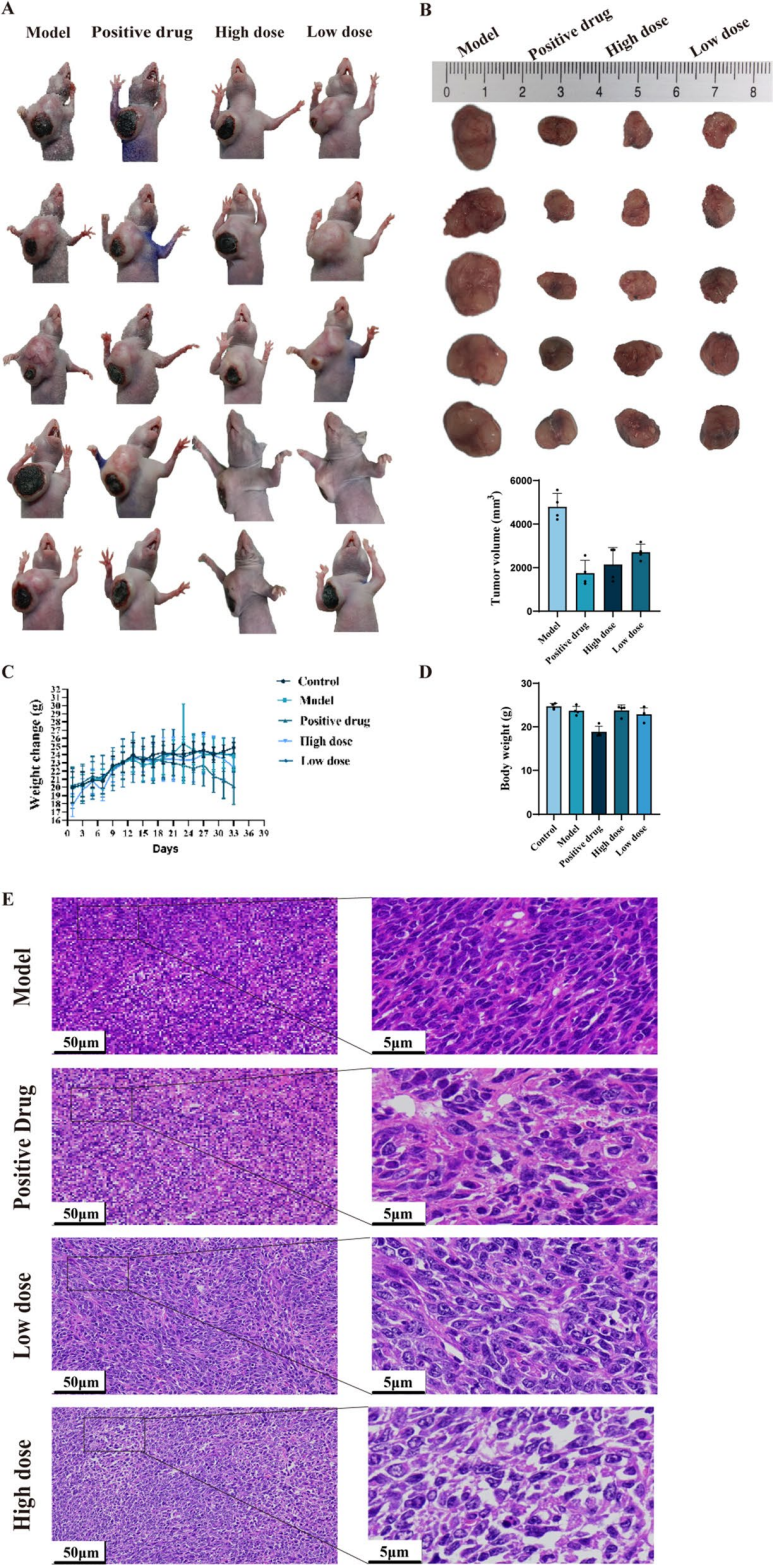
Final tumor size in each group of mice (**A**). Quantitative analysis of final tumor size and tumor volume in each group of mice (**B**). Body weight changes in mice over a five-week period. Mice were fasted the day before dissection, resulting in weight loss (**C**). Solid tumor weights in each group of mice (**D**). Observation of HE staining of mouse tumor tissues (**E**)

The 4T1 breast cancer nude mouse model was utilized, with administration of high-dose and low-dose TVEAE and positive control. Body weight monitoring demonstrated stable growth across all groups, indicating no significant systemic toxicity of TVEAE. After 21 days of treatment, TVEAE significantly inhibited tumor growth (Fig. 2A–E). The tumor inhibition rates in the low-dose group and high-dose group were 43.5% (*p* < 0.01) and 55.3% (*p* < 0.01), respectively, showing a dose-dependent relationship (r = 0.92, *p* < 0.001).

### Toxicological effects of TVEAE on hepatic and tumor tissues in murine models

**Fig.3:**
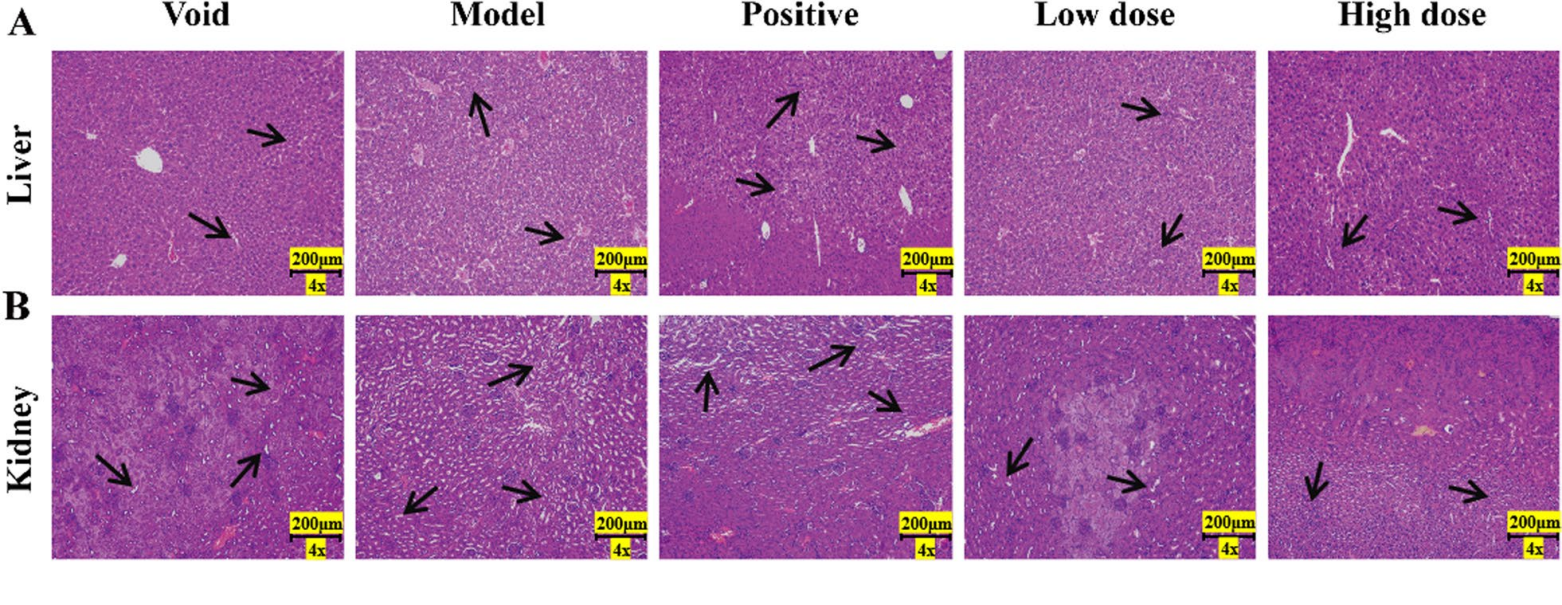
Observation of HE staining of mouse liver tissue (**A**). Observation of HE staining of mouse kidney tissue (**B**)

To further evaluate the impact of TVEAE on tumor growth, the researchers analyzed tissue samples from 4T1 xenograft models, including tumors, livers and kidneys. Observations by H&E staining revealed that TVEAE led to a looser arrangement of tumor cells and significantly enlarged areas of necrosis compared to controls. Although TVEAE produced some histopathological changes in the liver and kidney, it was still less toxic than the positive control drug (Fig. 3A, B).

### TVEAE inhibits TNBC by inducing ferroptosis

**Fig.4:**
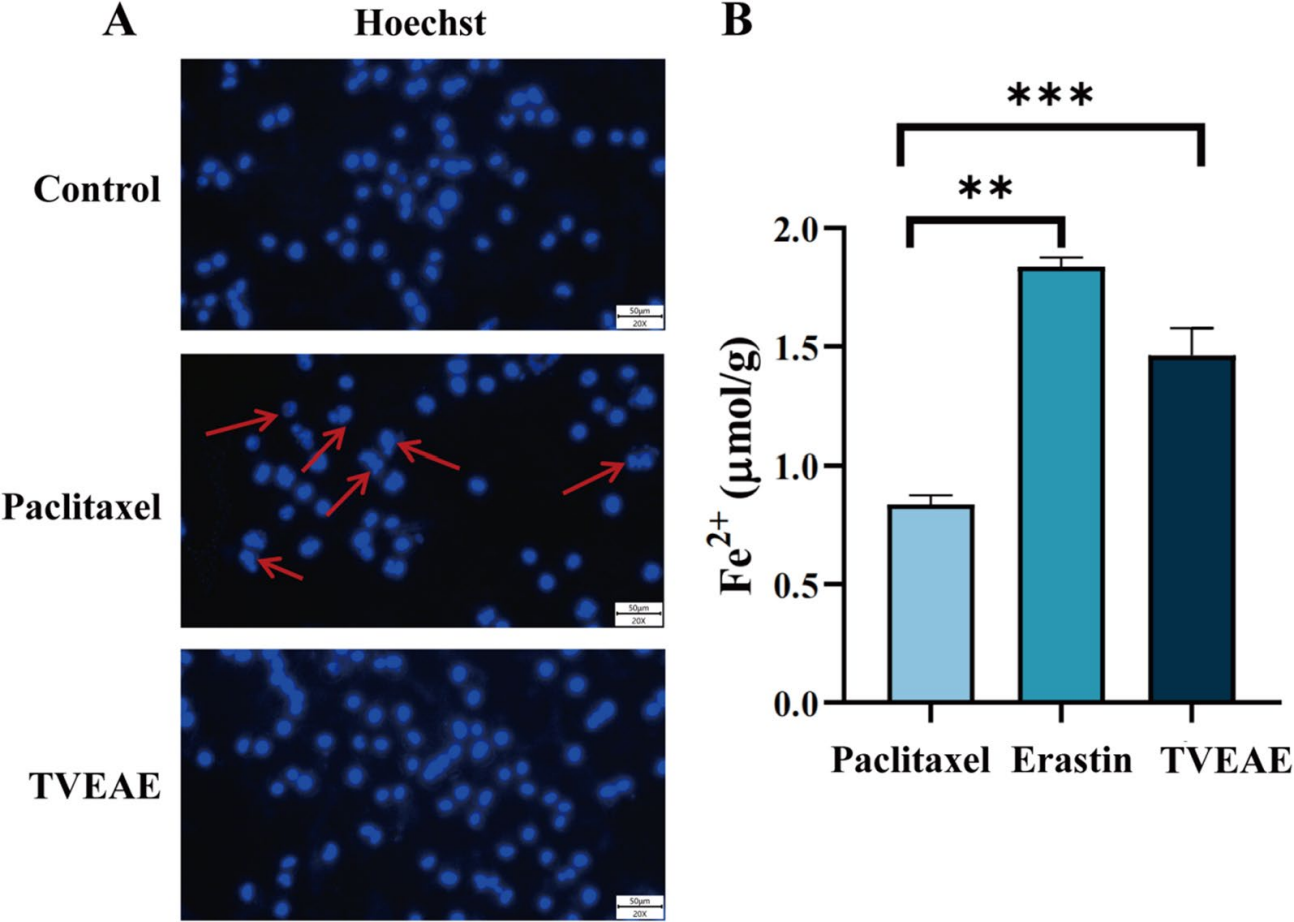
Hoechst 33,342 staining microscope image results (**A**). Histogram of ferrous ion detection results, displaying the intracellular ferrous ion content, expressed as the mean ± standard deviation of three independent experiments (**B**). *p < 0.05, **p < 0.01, ***p < 0.001, ****p < 0.0001 (n = 3 per group, where n represents three technical replicates in a single experiment)

**Fig.5:**
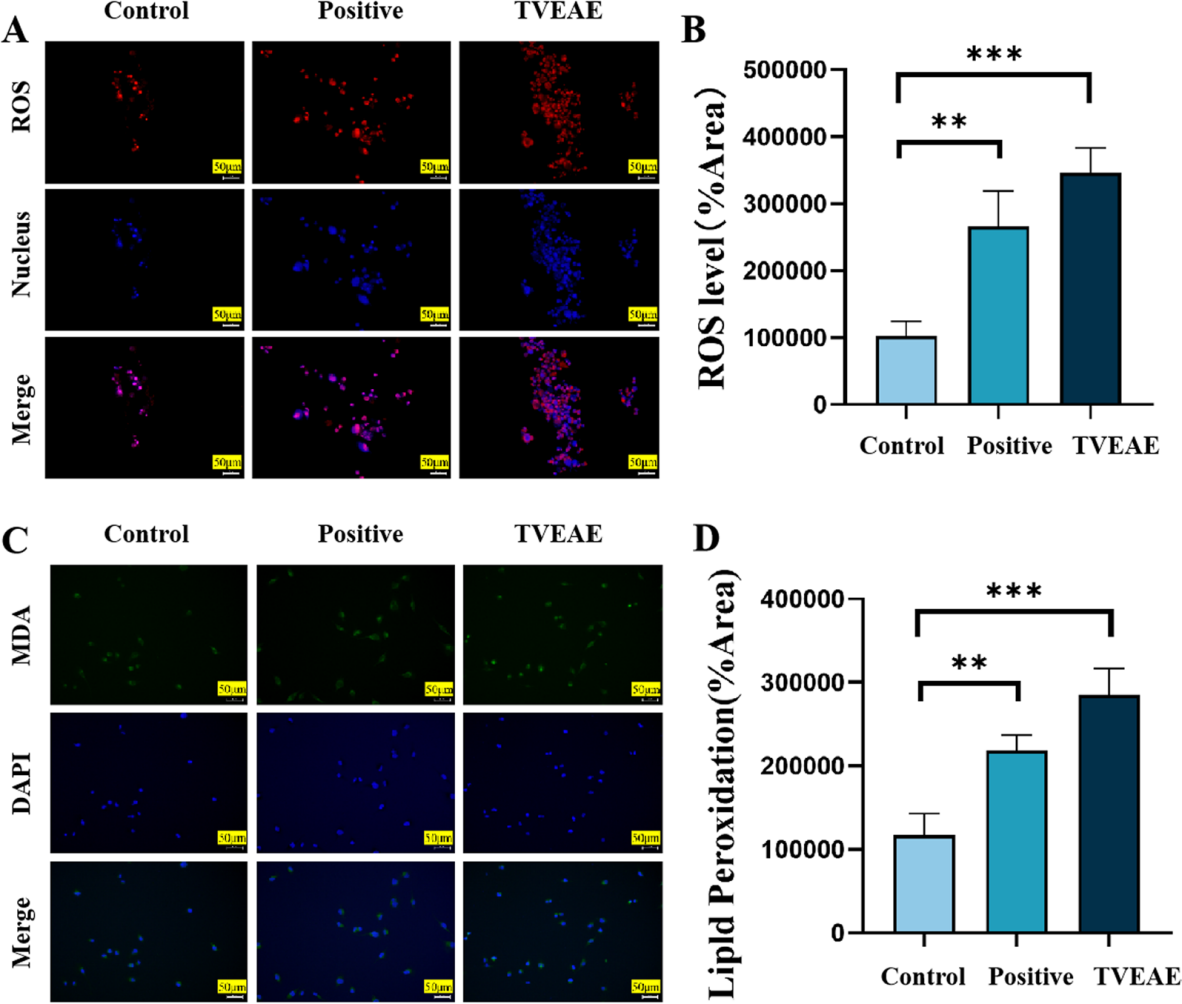
ROS staining microscopy image results (**A**). Quantification of relative fluorescence intensity assay (**B**). Microscopic image results of lipid peroxidation staining (**C**). Quantification of relative fluorescence intensity assay (**D**). *p < 0.05, **p < 0.01, ***p < 0.001, ****p < 0.0001 (n = 3 per group, where n represents three technical replicates in a single experiment)

In vivo and in vitro experimental model results showed that TVEAE had a significant inhibitory effect on TNBC. To systematically analyze the molecular mechanism by which TVEAE induces TNBC cell death. In this experiment, Hoechst 33,342 staining was used to observe the morphological characteristics of nuclear apoptosis. The level of ferrous ions was detected using a ferrous ion kit. The purpose is to explore the mechanisms of two programmed cell death pathways: apoptosis and ferroptosis. The analysis of Hoechst staining results showed (Fig. 4A) that the observed cell death was not a typical morphological feature of apoptosis. Therefore, it is suggested that TVEAE inhibits TNBC through apoptosis. The detection results of ferrous ion content showed (Fig. 4B) that ferrous ions accumulated after TVEAE administration treatment. Therefore, it is preliminarily indicated that TVEAE inhibits TNBC by inducing ferroptosis. To further investigate whether TVEAE induces ferroptosis in cells. In this study, ROS staining experiments and lipid peroxidation staining experiments were conducted. The experimental results showed that the lipid peroxidation level and ROS level of TVEAE administration group were significantly higher than those of the control group (Fig. 5A–D). This result proved that TVEAE inhibited TNBC by inducing ferroptosis.

### Go and KEGG pathway enrichment

**Fig.6:**
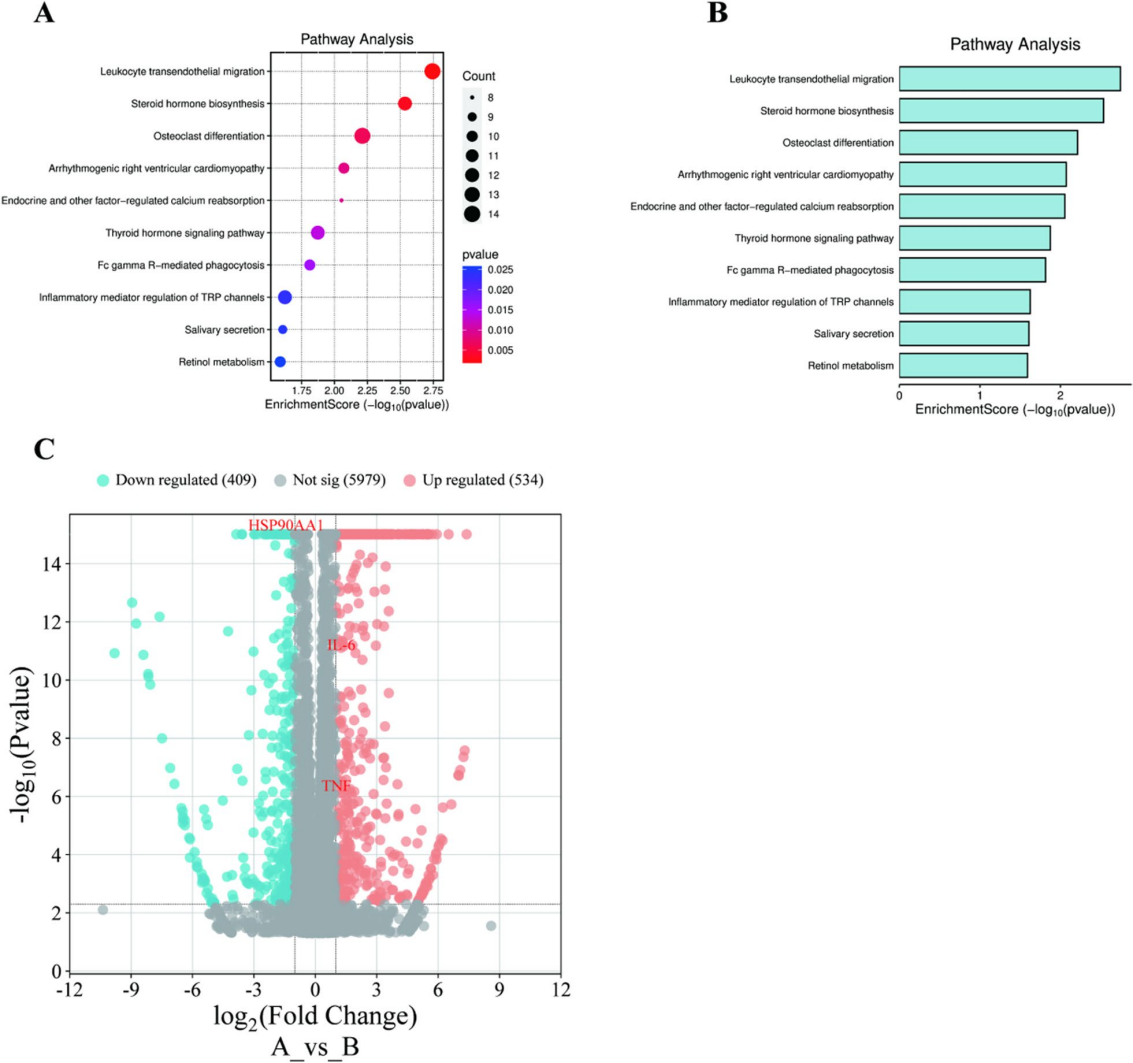
Bubble plot of KEGG analysis results of differentially expressed genes (**A**). Histogram of KEGG analysis results of differentially expressed genes (**B**). Volcanic diagram displays the analysis results of differentially expressed genes (**C**)

Based on the above results, in order to further explore the mechanism of action of TVEAE in inducing ferroptosis in TNBC cells we performed transcriptome analysis. The upstream transcriptome data were first uploaded to Wei Sheng Xin for DEseq2 based differential analysis. Subsequently, the differentially expressed genes identified were subjected to GO and KEGG pathway enrichment analysis. GO enrichment analysis revealed 249 significantly enriched biological processes. Its core target function is mainly related to granulocyte migration. The results of KEGG pathway enrichment showed 311 possible enriched signaling pathways (Fig. 6A). Among them, the leukocyte transendothelial migration pathway has the highest credibility (Fig. 6B *p* < 0.01). A total of 409 significantly down-regulated and 534 significantly up-regulated genes were identified by DESeq2 difference analysis (Fig. 6C).

### Identification of core therapeutic targets

**Fig.7:**
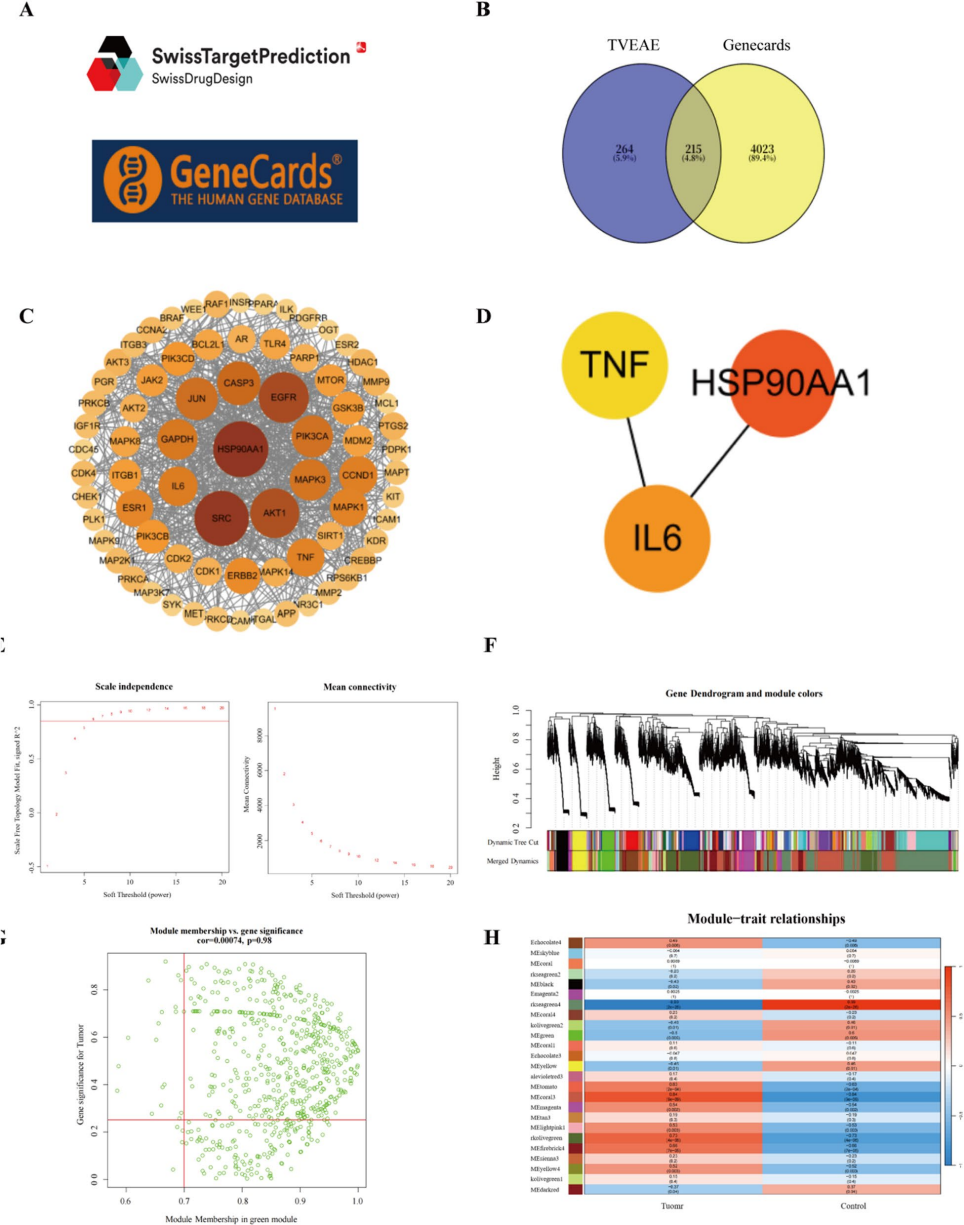
Swiss TargetPrediction Database and Geneclads Database (**A**). Venn diagram of TVEAE-TNBC target (**B**). PPI network diagram of involved targets of compound-disease intersection pathway (**C**). Intersection of WGCNA analysis results with CytoNCA algorithm results Gene (**D**). Scale independence analysis and mean connectivity of soft-threshold power from 1 to 20 (**E**). Clustering dendrograms of the different module genes (**F**). Scatter plots of correlations with respect to the green module genes (**G**). The correlation heat map between module genes and clinical traits (**H**)

In order to investigate the specific targets of TVEAE on TNBC, this study performed network pharmacology prediction and WGCNA. The Swiss TargetPrediction database provided a total of 786 potential targets for TVEAE components (Fig. 7A). A total of 18,389 BC targets and 4238 TNBC targets were identified from the GeneCards database. Among them, there are 4023 common cross-targets between BC and TNBC. Venn analysis pinpointed 201 overlapping targets between TVEAE and TNBC (Fig. 7B), indicating potential therapeutic targets. The protein–protein interaction (PPI) network of TVEAE therapeutic targets in TNBC was constructed using the search tool for recurring instances of neighbouring genes (STRING) database (Fig. 7C and Fig. S1). Using Cytoscape Network Analyzer (CytoNCA) algorithm to screen the top 5 targets based on degree values and taking the intersection of WGCNA analysis results (Fig. 7E–H and Fig. S2), the three most credible targets were identified, namely IL-6, TNF-*α*, and HSP90AA1 (Fig. 7D).

### TVEAE induces ferroptosis in TNBC via ICAM-1/GPX4 axis mediated by leukocyte transendothelial migration pathway

**Fig.8:**
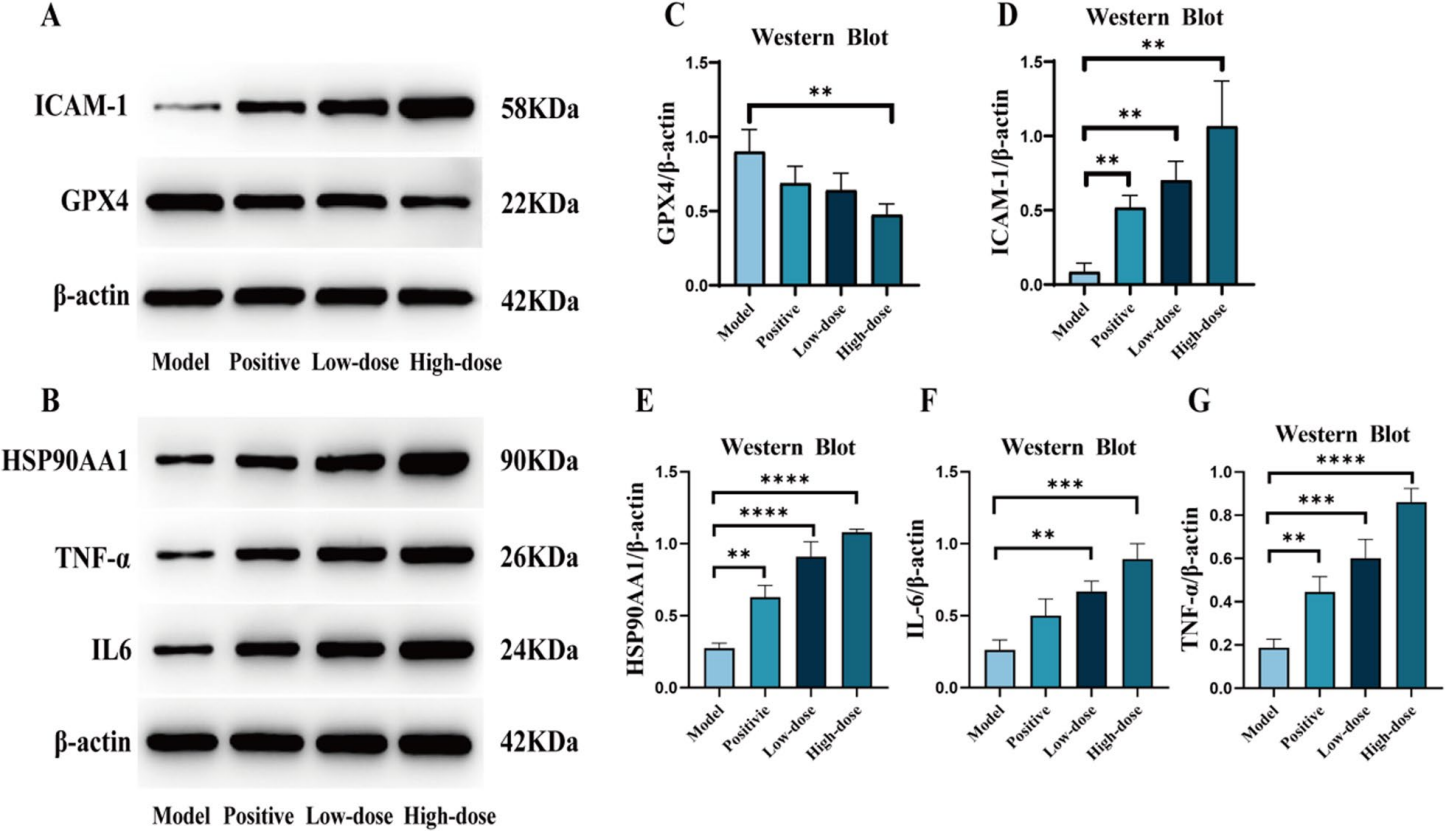
Western blot analysis bands. The effect of TVEAE on the expression of ICAM-1, GPX4, HSP90AA1, IL6, and TNF-*α* in tumor tissues (**A**, **B**). Display quantitative levels of ICAM-1, GPX4, HSP90AA1, IL6, and TNF-*α* (**C**–**G**). The results are expressed as mean ± SD, *p < 0.05, **p < 0.01, ***p < 0.001, ****p < 0.0001 (n = 3 per group, where n denotes three independent biological replicates)

In this study, we found that the signal pathway of leukocyte transendothelial migration in TNBC was significantly activated by transcriptome pathway enrichment analysis. Mechanistically, ICAM-1, a key effector of this pathway, suppressed TNBC progression by downregulating GPX4 and thereby inducing ferroptosis. Prior studies indicate that inflammation-related factors such as TNF-*α*, IL-6, and HSP90AA1 can regulate ICAM-1 by activating the NF-κB signaling pathway. To validate this axis, we assessed protein levels of TNF-*α*, IL-6, HSP90AA1, ICAM-1, and GPX4 in TNBC models by Western blotting. The experimental results demonstrated that TVEAE treatment significantly increased TNF-*α*, IL-6, HSP90AA1, and ICAM-1 while downregulating GPX4 (Fig. 8A–G). These findings indicate that TVEAE may induce ferroptosis in TNBC cells through multi-target regulation of the leukocyte transendothelial migration pathway.

### Identification of potential anti-TNBC active compounds

**Fig.9:**
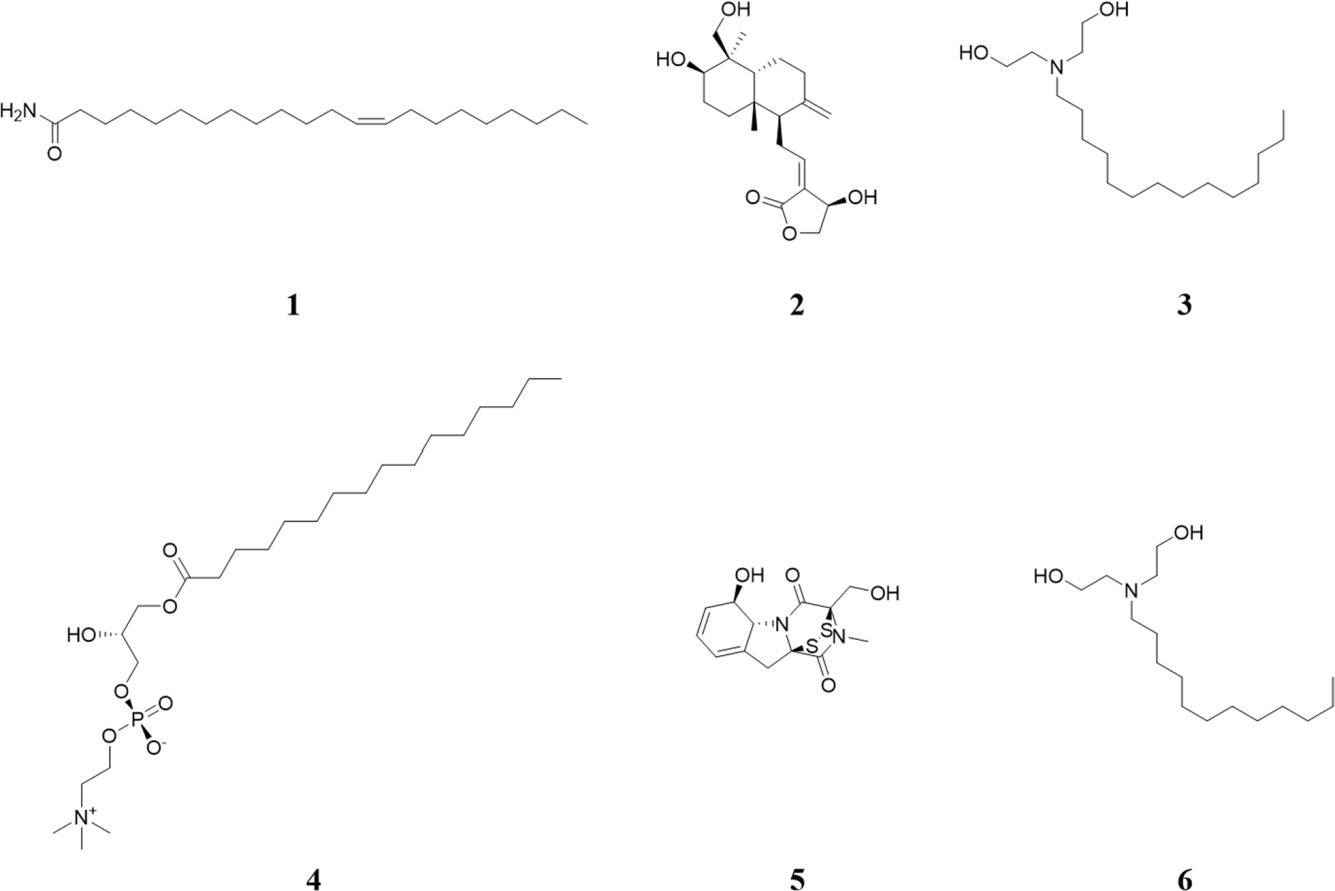
The potential active compounds identified by molecular network

Molecular networking analysis generated a batch of candidate compounds, from which eighteen were successfully annotated (Fig. S3). Subsequently, six potential bioactive compounds were identified by analyzing the retention time, high-resolution mass-to-charge ratio (*m/z*) signals, and ESI-MS2 fragmentation patterns (Table S1 and Fig. S4), including N-lauryldiethanolamine (1541-67-9), erucamide (112-84-5), Gliotoxin (67-99-2) (Fig. 9).

Results from molecular docking and binding energy calculations showed that erucamide exhibits the most favorable binding energy with the TNF-*α* protein, with a binding energy of − 50.1925 kJ/mol. The binding energy values for the interactions between the other identified compounds and each target protein are presented in Table 1. Notably, all six candidate compounds exhibit strong binding affinity with HSP90AA1, IL6, and TNF-*α*, which confirms the stable interactions between TVEAE and HSP90AA1, IL6, and TNF-*α* (Fig. S5).

**Table 1:** The binding energy values between compound and different proteins

Compound	Protein	PDB ID	Binding Energy KJ/mol
Erucamide	TNF-*α*	7KP7	− 50.1925
	HSP90AA1	7D1V	− 11.3579
Andropanolide	TNF-*α*	7KP7	− 36.5731
	IL6	2HMH	− 21.8856
	HSP90AA1	7D22	− 14.2836
Tetradecyldiethanolamine	TNF-*α*	7KP7	− 31.8663
	HSP90AA1	7D24	− 19.8763
	IL6	2HMH	− 9.7698
LPC(16:0/0:0)	HSP90AA1	7D24	− 16.2674
	IL6	2HMH	− 15.4264
Gliotoxin	IL6	2HMH	− 19.1006
	HSP90AA1	7D25	− 15.0473
Lauryldiethanolamine	IL6	2HMH	− 18.5477
	HSP90AA1	7D22	− 16.3264
	TNF-*α*	7KP8	− 14.079

## Discussion

In this study, TVEAE, a secondary metabolite isolated from the endophytic fungus *Trichoderma viride* of *Coreopsis basalis*, demonstrated significant anti-TNBC activity in both in vitro and in vivo experiments, effectively inhibiting tumor cell proliferation and suppressing metastasis. However, our understanding of its mechanisms and targets is remains unclear. This has significantly impeded its development as a candidate anticancer drug. Further investigation revealed that TVEAE’s key bioactive compounds were found to notably increase the levels of intracellular Fe^2^⁺, ROS, and lipid peroxides in TNBC cells. Nevertheless, Hoechst 33,342 staining did not reveal any apoptotic morphology. These results indicate that ferroptosis is the predominant form of cell death induced by TVEAE.

Through network pharmacology, whole-transcriptome profiling, and bioinformatic, we demonstrated that TVEAE induces ferroptosis in TNBC via dysregulation of the TEM pathway. Leukocyte TEM, a critical step in inflammatory responses and immune surveillance, is tightly regulated by a clear molecular mechanism. Roland et al. experimentally showed that ARHGAP2 promotes leukocyte recruitment to inflammatory sites but also acts as a negative regulator of TEM [34]. Zhu et al. demonstrated that G-CSFR-mediated inflammatory signaling modulates leukocyte TEM via the ICAM1-PKC*α* axis [35]. Abdala-Valencia et al. provided the first experimental evidence that ERK signaling regulates VCAM-1-dependent TEM of leukocytes [36]. However, the therapeutic implications of this mechanism in TNBC remain largely unknown. Chronic inflammation and cancer progression are mechanistically linked through multiple pathways [37]. Here, we first report that in TNBC, TVEAE restricts tumor growth through blocking TEM signaling, thereby inducing a ferroptosis-dependent cell death process.

Furthermore, based on the results of this study, we speculated that ICAM-1 may serve as a therapeutic target in TNBC. ICAM-1 is a pivotal regulator of leukocyte TEM. Emerging evidence has implicated ICAM-1 in TNBC progression of TNBC, and multiple studies have delineated its functional mechanisms and clinical relevance. Peng et al. experimentally validated ICAM-1 as a dual-function target, demonstrating its utility as both a therapeutic vulnerability and a potential prognostic biomarker [38]. Ying et al. reported elevated ICAM-1 expression in TNBC, associating it to inflammatory responses, apoptosis modulation, and diverse oncogenic processes [39]. Notably, Kang et al. identified ICAM-1 as a crucial adaptor protein facilitating EGFR activation, thereby driving malignant progression [40]. Although ICAM-1 exerts tumor-suppressive effects, its involvement in ferroptosis-driven TNBC growth arrest remains elusive. Here, we identify TVEAE as a multi-targeted regulator that coordinates IL-6/TNF-*α*/HSP90AA1 inhibition to potentiate ICAM-1 signaling. This cascade induces mitochondrial ROS overproduction, glutathione (GSH) depletion, GPX4 inactivation, and lipid peroxidation accumulation, resulting in tumor cell ferroptosis. Wei et al. identified withanolide AC, a bioactive metabolite from *Tubocapsicum anomalum*, as a potent ferroptosis inducer that promotes GPX4 ubiquitination and autophagic degradation, suggesting it as a lead candidate for TNBC therapy [41].

Collectively, these findings demonstrate that the pharmacological induction of ferroptosis is a promising therapeutic strategy for TNBC. The low toxicity profile of natural products positions them as a potentially transformative therapeutic strategy for TNBC. A resorcinolic lactone isolated from the natural product *A. tataricus* strain *Ilyonectria* sp. functions as a novel and potent copper ionophore. By covalently targeting PRDX1 to induce cuproptosis, it exerts therapeutic effects against TNBC [42]. In this study, we identified erucamide, andropanolide, tetradecyldiethanolamine, LPC (16:0/0:0), gliotoxin and lauryldiethanolamine in TVEAE, which may play pivotal roles in its anti-TNBC effects. In summary, we establish for the first time that TVEAE has anti-TNBC effects in both animal and cellular systems. Mechanistically, TVEAE induces ferroptosis by simultaneously modulating key components of the leukocyte TEM signaling pathway.

## Supplementary Information

**Additional file 1.**

## Data Availability

All data generated and analyzed during this study are included in this published article and its supporting information.

## References

[1] Bianchini G, De Angelis C, Licata L, Gianni L. Treatment landscape of triple-negative breast cancer-expanded options, evolving needs. Nat Rev Clin Oncol. 2022;19(2):91–0113. 10.1038/s41571-021-00565-2. 34754128 10.1038/s41571-021-00565-2

[2] Borri F, Granaglia A. Pathology of triple negative breast cancer. Semin Cancer Biol. 2021;72:136–45. 10.1016/j.semcancer.2020.06.005. 32544511 10.1016/j.semcancer.2020.06.005

[3] Bai X, Ni J, Beretov J, Graham P, Li Y. Triple-negative breast cancer therapeutic resistance: where is the Achilles’ heel? Cancer Lett. 2021;497:100–11. 10.1016/j.canlet.2020.10.016. 33069769 10.1016/j.canlet.2020.10.016

[4] Asleh K, Riaz N, Nielsen TO. Heterogeneity of triple negative breast cancer: current advances in subtyping and treatment implications. J Exp Clin Cancer Res. 2022;41(1):265. 10.1186/s13046-022-02476-1. 36050786 10.1186/s13046-022-02476-1 PMC9434975

[5] Siegel RL, Miller KD, Fuchs HE, Jemal A. Cancer statistics, 2022. CA Cancer J Clin. 2022;72(1):7–33. 10.3322/caac.21708. 35020204 10.3322/caac.21708

[6] Yin L, Duan JJ, Bian XW, Yu SC. Triple-negative breast cancer molecular subtyping and treatment progress. Breast Cancer Res. 2020;22(1):61. 10.1186/s13058-020-01296-5. 32517735 10.1186/s13058-020-01296-5 PMC7285581

[7] Harbeck N, Gnant M. Breast cancer. Lancet. 2017;389(10074):1134–50. 10.1016/S0140-6736(16)31891-8. 27865536 10.1016/S0140-6736(16)31891-8

[8] Zhang J, Zhang Z, Huang Z, Li M, Yang F, Wu Z, et al. Isotoosendanin exerts inhibition on triple-negative breast cancer through abrogating TGF-*β*-induced epithelial-mesenchymal transition via directly targeting TGF*β*R1. Acta Pharm Sin B. 2023;13(7):2990–3007. 10.1016/j.apsb.2023.05.006. 37521871 10.1016/j.apsb.2023.05.006 PMC10372922

[9] Yang C, Liu H, Feng X, Shi H, Jiang Y, Li J, et al. Research hotspots and frontiers of neoadjuvant therapy in triple-negative breast cancer: a bibliometric analysis of publications between 2002 and 2023. Int J Surg. 2024;110(8):4976–92. 10.1097/JS9.0000000000001586. 39143709 10.1097/JS9.0000000000001586 PMC11326012

[10] Padzińska-Pruszyńska I, Kucharzewska P, Matejuk A, Górczak M, Kubiak M, Taciak B, et al. Macrophages: key players in the battle against triple-negative breast cancer. Int J Mol Sci. 2024;25(19):10781. 10.3390/ijms251910781. 39409110 10.3390/ijms251910781 PMC11476577

[11] Xu L, Xu P, Wang J, Ji H, Zhang L, Tang Z. Advancements in clinical research and emerging therapies for triple-negative breast cancer treatment. Eur J Pharmacol. 2025;988:177202. 10.1016/j.ejphar.2024.177202. 39675457 10.1016/j.ejphar.2024.177202

[12] Sun K, Zhang B, Lei S, Zheng R, Liang X, Li L, et al. Incidence, mortality, and disability-adjusted life years of female breast cancer in China, 2022. Chin Med J. 2024;137(20):2429–36. 10.1097/CM9.0000000000003278. 39238088 10.1097/CM9.0000000000003278 PMC11479498

[13] Ji YT, Liu SW, Zhang YM, Duan HY, Liu XM, Feng ZW, et al. Comparison of the latest cancer statistics, cancer epidemic trends and determinants between China and the United States. Zhonghua Zhong Liu Za Zhi. 2024;46(7):646–56. 10.3760/cma.j.cn112152-20240208-00068. 38764329 10.3760/cma.j.cn112152-20240208-00068

[14] Zhu S, Wu Y, Song B, Yi M, Yan Y, Mei Q, et al. Recent advances in targeted strategies for triple-negative breast cancer. J Hematol Oncol. 2023;16(1):100. 10.1186/s13045-023-01497-3. 37641116 10.1186/s13045-023-01497-3 PMC10464091

[15] Villacampa G, Navarro V, Matikas A, Ribeiro JM, Schettini F, Tolosa P, et al. Neoadjuvant immune checkpoint inhibitors plus chemotherapy in early breast cancer: a systematic review and meta-analysis. JAMA Oncol. 2024;10(10):1331–41. 10.1001/jamaoncol.2024.3456. 39207778 10.1001/jamaoncol.2024.3456 PMC12422158

[16] Maughan KL, Lutterbie MA, Ham PS. Treatment of breast cancer. Am Fam Physician. 2010;81(11):1339–46. 20521754

[17] Woolston C. Breast cancer: 4 big questions. Nature. 2015;527(7578):S120. 10.1038/527S120a. 26580163 10.1038/527S120a

[18] Mei D, Chen B, He B, Liu H, Lin Z, Lin J, et al. Actively priming autophagic cell death with novel transferrin receptor-targeted nanomedicine for synergistic chemotherapy against breast cancer. Acta Pharm Sin B. 2019;9(5):1061–77. 10.1016/j.apsb.2019.03.006. 31649854 10.1016/j.apsb.2019.03.006 PMC6804482

[19] Liao M, Qin R, Huang W, Zhu HP, Peng F, Han B, et al. Targeting regulated cell death (RCD) with small-molecule compounds in triple-negative breast cancer: a revisited perspective from molecular mechanisms to targeted therapies. J Hematol Oncol. 2022;15(1):44. 10.1186/s13045-022-01260-0. 35414025 10.1186/s13045-022-01260-0 PMC9006445

[20] Zhang J, Wu Y, Li Y, Li S, Liu J, Yang X, et al. Natural products and derivatives for breast cancer treatment: from drug discovery to molecular mechanism. Phytomedicine. 2024;129:155600. 10.1016/j.phymed.2024.155600. 38614043 10.1016/j.phymed.2024.155600

[21] Yang C, Deng X, Tang Y, Tang H, Xia C. Natural products reverse cisplatin resistance in the hypoxic tumor microenvironment. Cancer Lett. 2024;598:217116. 10.1016/j.canlet.2024.217116. 39002694 10.1016/j.canlet.2024.217116

[22] Sun J, Zhan X, Wang W, Yang X, Liu Y, Yang H, et al. Natural aporphine alkaloids: a comprehensive review of phytochemistry, pharmacokinetics, anticancer activities, and clinical application. J Adv Res. 2024;63:231–53. 10.1016/j.jare.2023.11.003. 37935346 10.1016/j.jare.2023.11.003 PMC11380034

[23] Deng R, Zong GF, Wang X, Yue BJ, Cheng P, Tao RZ, et al. Promises of natural products as clinical applications for cancer. Biochimica et Biophysica Acta Rev Cancer. 2025;1880(1):189241. 10.1016/j.bbcan.2024.189241. 10.1016/j.bbcan.2024.189241 39674416

[24] Chen W, Zhou W, Liu S. The key role of natural products in the fight against endometrial Cancer. Int Immunopharmacol. 2025;151:114344. 10.1016/j.intimp.2025.114344. 40015208 10.1016/j.intimp.2025.114344

[25] Mayrhofer BF, Iantas J, Noriler SA, Ponomareva LV, Thorson JS, Rohr J, et al. Highly diverse endophytic fungi from Serra do Amolar-Pantanal (Brazil) producing bioactive secondary metabolites against phytopathogens. Front Microbiol. 2024;15:1501182. 10.3389/fmicb.2024.1501182. 39777144 10.3389/fmicb.2024.1501182 PMC11703833

[26] Wu J, Ye J, Cen J, Chen Y, Xu J. Induction of three new secondary metabolites by the co-culture of endophytic fungi *Phomopsis asparagi* DHS-48 and *Phomopsis* sp. DHS-11 isolated from the Chinese mangrove plant *Rhizophora mangle*. Mar Drugs. 2024;22(8):332. 10.3390/md22080332. 39195448 10.3390/md22080332 PMC11355877

[27] El-Zehery HRA, Ashry NM, Faiesal AA, Attia MS, Abdel-Maksoud MA, El-Tayeb MA, et al. Antibacterial and anticancer potential of bioactive compounds and secondary metabolites of endophytic fungi isolated from *Anethum graveolens*. Front Microbiol. 2024;15:1448191. 10.3389/fmicb.2024.1448191. 39435441 10.3389/fmicb.2024.1448191 PMC11491383

[28] Cao LL, Gao ZJ, Wang DX, Nie Y, Yu H, Zhang P. Aspertaichamide B, a new anti-tumor prenylated indole alkaloid from the fungus *Aspergillus japonicus* TE-739D. Appl Microbiol Biotechnol. 2024;108(1):473. 10.1007/s00253-024-13313-0. 39320549 10.1007/s00253-024-13313-0 PMC11424712

[29] Wufuer Y, Yang X, Guo L, Aximujiang K, Zhong L, Yunusi K, et al. The antitumor effect and mechanism of total flavonoids from *Coreopsis tinctoria* Nutt on lung cancer using network pharmacology and molecular docking. Front Pharmacol. 2022;13:761785. 10.3389/fphar.2022.761785. 35350758 10.3389/fphar.2022.761785 PMC8957955

[30] Mou Y, Wang J, Wu J, He D, Zhang C, Duan C, et al. Ferroptosis, a new form of cell death: opportunities and challenges in cancer. J Hematol Oncol. 2019;12(1):34. 10.1186/s13045-019-0720-y. 30925886 10.1186/s13045-019-0720-y PMC6441206

[31] Sun S, Shen J, Jiang J, Wang F, Min J. Targeting ferroptosis opens new avenues for the development of novel therapeutics. Signal Transduct Target Ther. 2023;8(1):372. 10.1038/s41392-023-01606-1. 37735472 10.1038/s41392-023-01606-1 PMC10514338

[32] Luo Y, Bai XY, Zhang L, Hu QQ, Zhang N, Cheng JZ, et al. Ferroptosis in cancer therapy: mechanisms, small molecule inducers, and novel approaches. Drug Des Devel Ther. 2024;18:2485–529. 10.2147/DDDT.S472178. 38919962 10.2147/DDDT.S472178 PMC11198730

[33] Zhang HL, Hu BX, Ye ZP, Li ZL, Liu S, Zhong WQ, et al. TRPML1 triggers ferroptosis defense and is a potential therapeutic target in AKT-hyperactivated cancer. Sci Transl Med. 2024;16(753):eadk0330. 10.1126/scitranslmed.adk0330. 38924427 10.1126/scitranslmed.adk0330

[34] Csépányi-Kömi R, Wisniewski É, Bartos B, Lévai P, Németh T, Balázs B, et al. Rac GTPase activating protein ARHGAP25 regulates leukocyte transendothelial migration in mice. J Immunol. 2016;197(7):2807–15. 10.4049/jimmunol.1502342. 27566826 10.4049/jimmunol.1502342

[35] Zhu Z, Ling X, Wang G, Xie J. G-CSFR-induced leukocyte transendothelial migration during the inflammatory response is regulated by the ICAM1-PKCa axis: based on multiomics integration analysis. Cell Biol Toxicol. 2024;40(1):90. 10.1007/s10565-024-09934-w. 39433604 10.1007/s10565-024-09934-w PMC11493794

[36] Abdala-Valencia H, Berdnikovs S, Cook-Mills JM. Mechanisms for vascular cell adhesion molecule-1 activation of ERK1/2 during leukocyte transendothelial migration. PLoS ONE. 2011;6(10):e26706. 10.1371/journal.pone.0026706. 22031842 10.1371/journal.pone.0026706 PMC3198778

[37] Greten FR, Grivennikov SI. Inflammation and cancer: triggers, mechanisms, and consequences. Immunity. 2019;51(1):27–41. 10.1016/j.immuni.2019.06.025. 31315034 10.1016/j.immuni.2019.06.025 PMC6831096

[38] Guo P, Huang J, Wang L, Jia D, Yang J, Dillon DA, et al. ICAM-1 as a molecular target for triple negative breast cancer. Proc Natl Acad Sci U S A. 2014;111(41):14710–5. 10.1073/pnas.1408556111. 25267626 10.1073/pnas.1408556111 PMC4205631

[39] Zhang Y, Fan J, Wang X, Wu Z, Ma W, Ma B. Role of ICAM-1 in triple-negative breast cancer. Open Med. 2024;19(1):20240969. 10.1515/med-2024-0969. 10.1515/med-2024-0969 PMC11117456 38799250

[40] Kang JH, Uddin N, Kim S, Zhao Y, Yoo KC, Kim MJ, et al. Tumor-intrinsic role of ICAM-1 in driving metastatic progression of triple-negative breast cancer through direct interaction with EGFR. Mol Cancer. 2024;23(1):230. 10.1186/s12943-024-02150-4. 39415210 10.1186/s12943-024-02150-4 PMC11481280

[41] Chen YM, Xu W, Liu Y, Zhang JH, Yang YY, Wang ZW, et al. Anomanolide C suppresses tumor progression and metastasis by ubiquitinating GPX4-driven autophagy-dependent ferroptosis in triple negative breast cancer. Int J Biol Sci. 2023;19(8):2531–50. 10.7150/ijbs.82120. 37215985 10.7150/ijbs.82120 PMC10197885

[42] Feng L, Wu TZ, Guo XR, Wang YJ, Wang XJ, Liu SX, et al. Discovery of natural resorcylic acid lactones as novel potent copper ionophores covalently targeting PRDX1 to induce cuproptosis for triple-negative breast cancer therapy. ACS Cent Sci. 2025;11(2):357–70. 10.1021/acscentsci.4c02188. 40028362 10.1021/acscentsci.4c02188 PMC11869127

