# Supplementary material for: The ethyl acetate extract from Trichoderma viride fermentation acts by downregulating the leukocyte transendothelial migration signaling pathway to induce ferroptosis in triple-negative breast cancer cells
Source: Nat Prod Bioprospect. 2026 Jan 10;16(1):15. doi: 10.1007/s13659-025-00569-w (PMC12790549; doi:10.1007/s13659-025-00569-w)
Supplement: Supplementary file 1 — Additional file 1. [file 13659_2025_569_MOESM1_ESM.docx]

**Supporting Information**

**The ethyl acetate extract from *Trichoderma viride* fermentation acts by downregulating the leukocyte transendothelial migration signaling pathway to induce ferroptosis in triple-negative breast cancer cells**

Yu Kuang^a #^, Bai-Hui Lu^a #^, Jia-Yi Wu^a^, Song-Yu Wu^a^, Hai-Yan Fu^a^, Qing-Yan Nan^c^, Jing Li^a^ *, Xiao-Long Yang^a, b^ *

*^a^ School of Pharmaceutical Sciences, South-Central Minzu University, Wuhan, 430074, China*

*^b^ School of Pharmacy, Anhui University of Chinese Medicine, Hefei, 230012, China*

*^c^ College of Life Sciences, South-Central Minzu University, Wuhan 430074, China*

**^#^** These authors contributed equally

***** Corresponding author.

Jing Li, E-mail: [jinglisxu@163.com](mailto:jinglisxu@163.com).

Xiaolong Yang, E-mail: yxl19830915@163.com.

**Content**

[Figure S1. PPI network built by Cytoscape. 3](#_Toc19608)

[Figure S2. Diagram of WGCNA Result Analysis. 3](#_Toc184)

[Figure S3. Compounds 1-18 identified by molecular networking technology 4](#_Toc31580)

[Figure S4. Mass Range obtained from the LC-MS of TVEAE. 6](#_Toc26774)

[Figure S5. Diagram of Molecular Docking Results 7](#_Toc25314)

[Table S1. Chemical constituents identified of TVEAE 10](#_Toc25028)

**Figure S1.** PPI network built by Cytoscape.

**
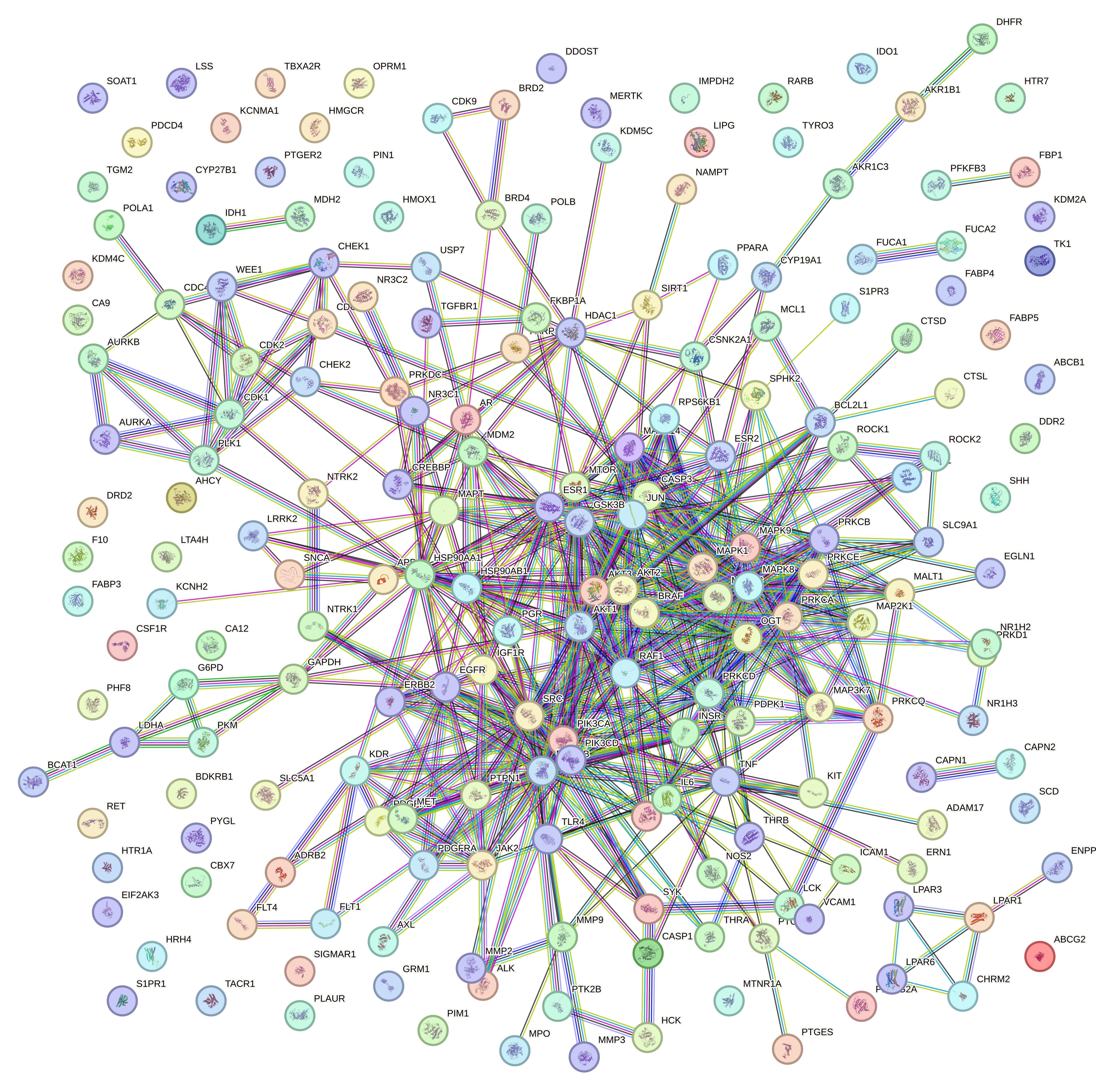
**

**Figure S2.** Diagram of WGCNA Result Analysis. (A) Checking the scale-free topology using soft-thresholds with β ＝ 7. (B) Gene Module Clustering Results Diagram. (C) Identifying key modules significantly associated with tumors. (D) Visualize and verify the quality of module division, and display the network connection structures within and between modules.


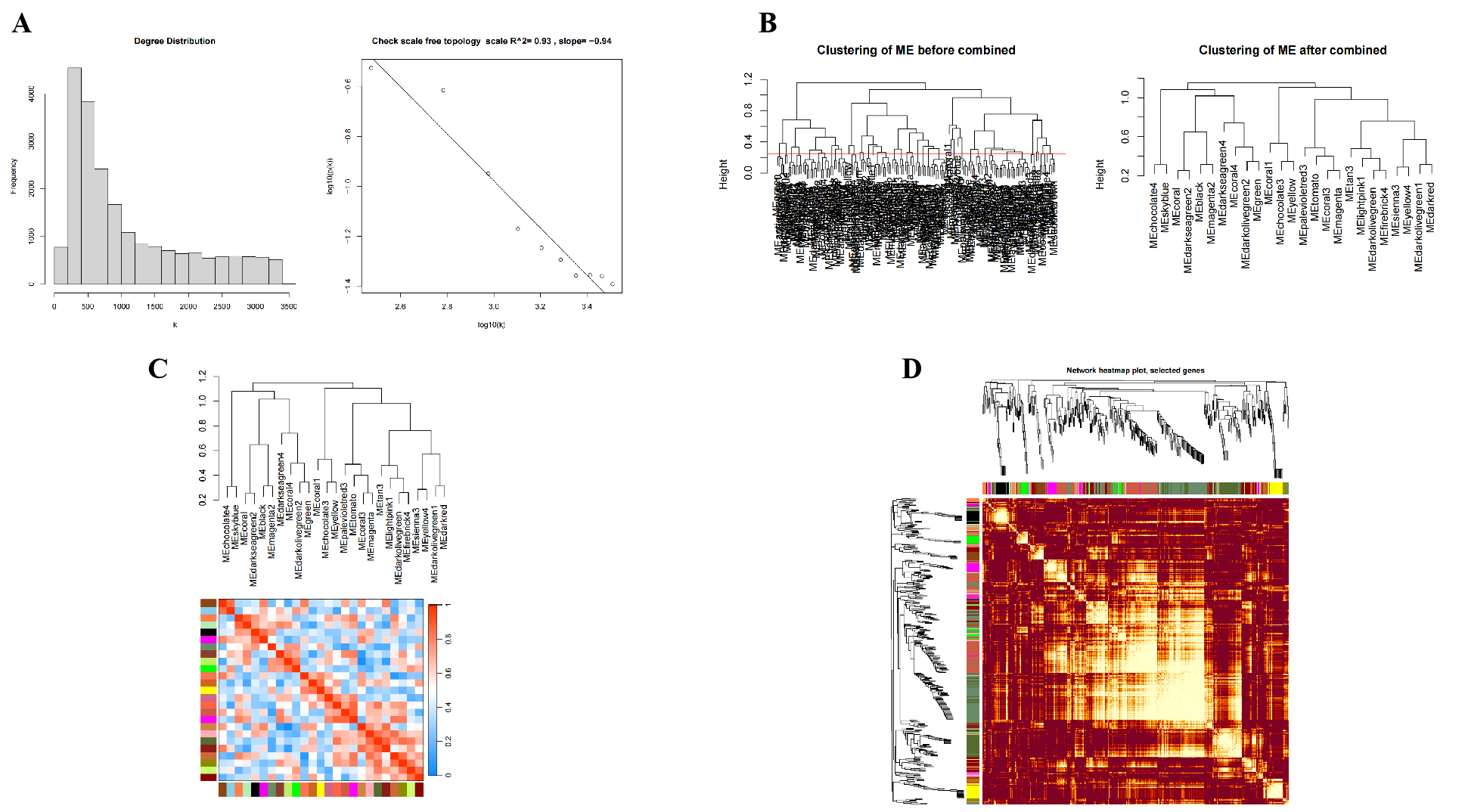


**Figure S3.** Compounds **1-18** identified by molecular networking technology.


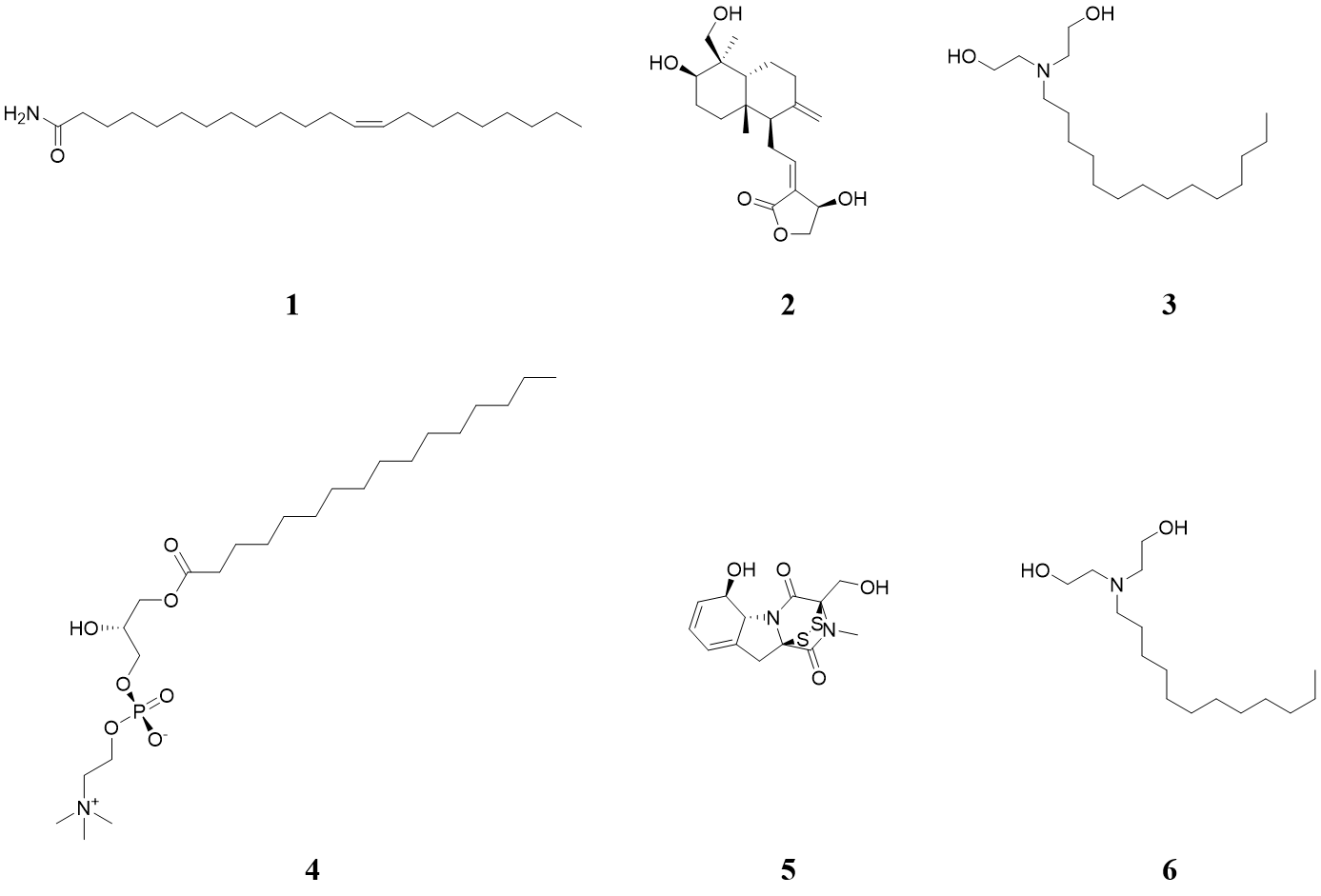

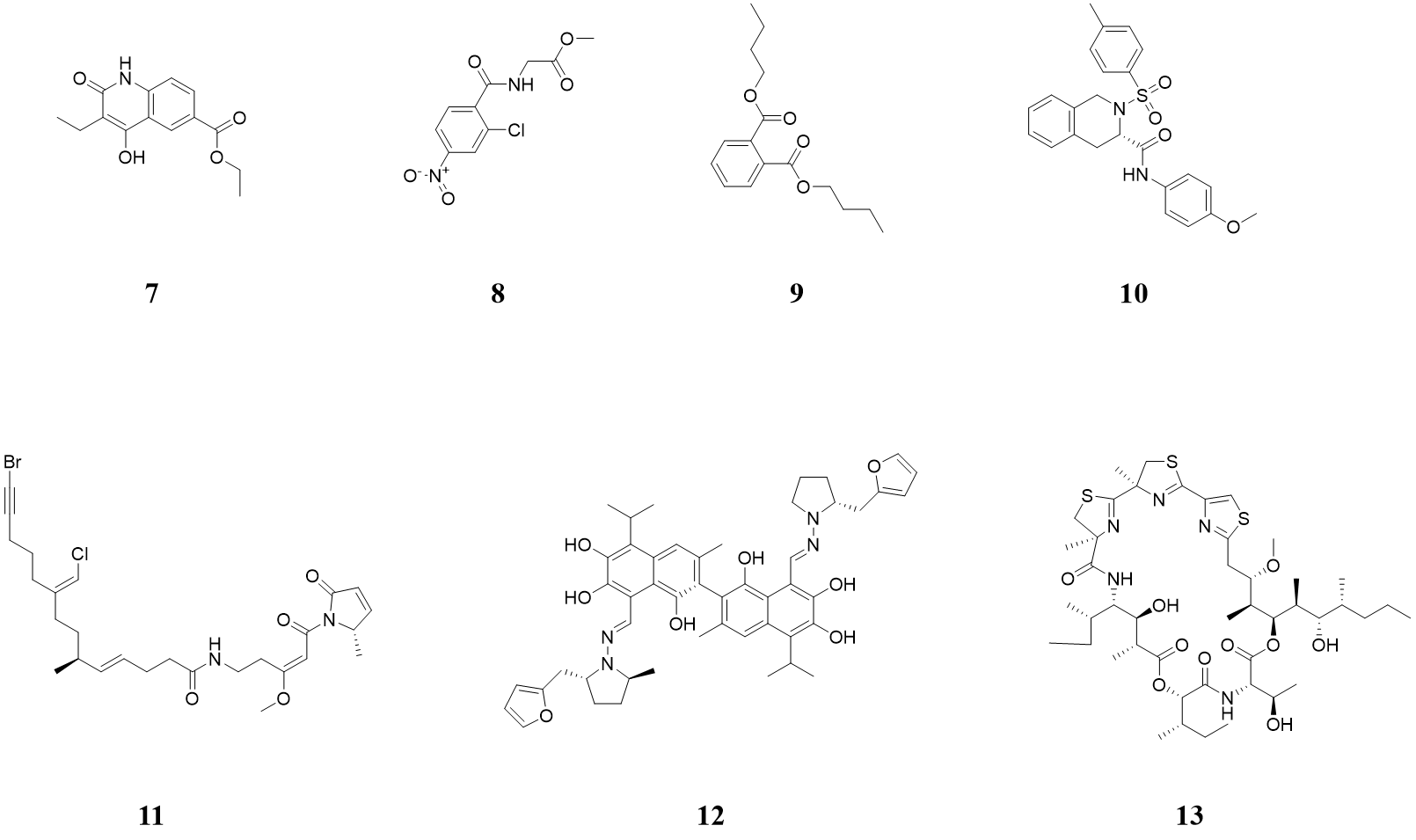


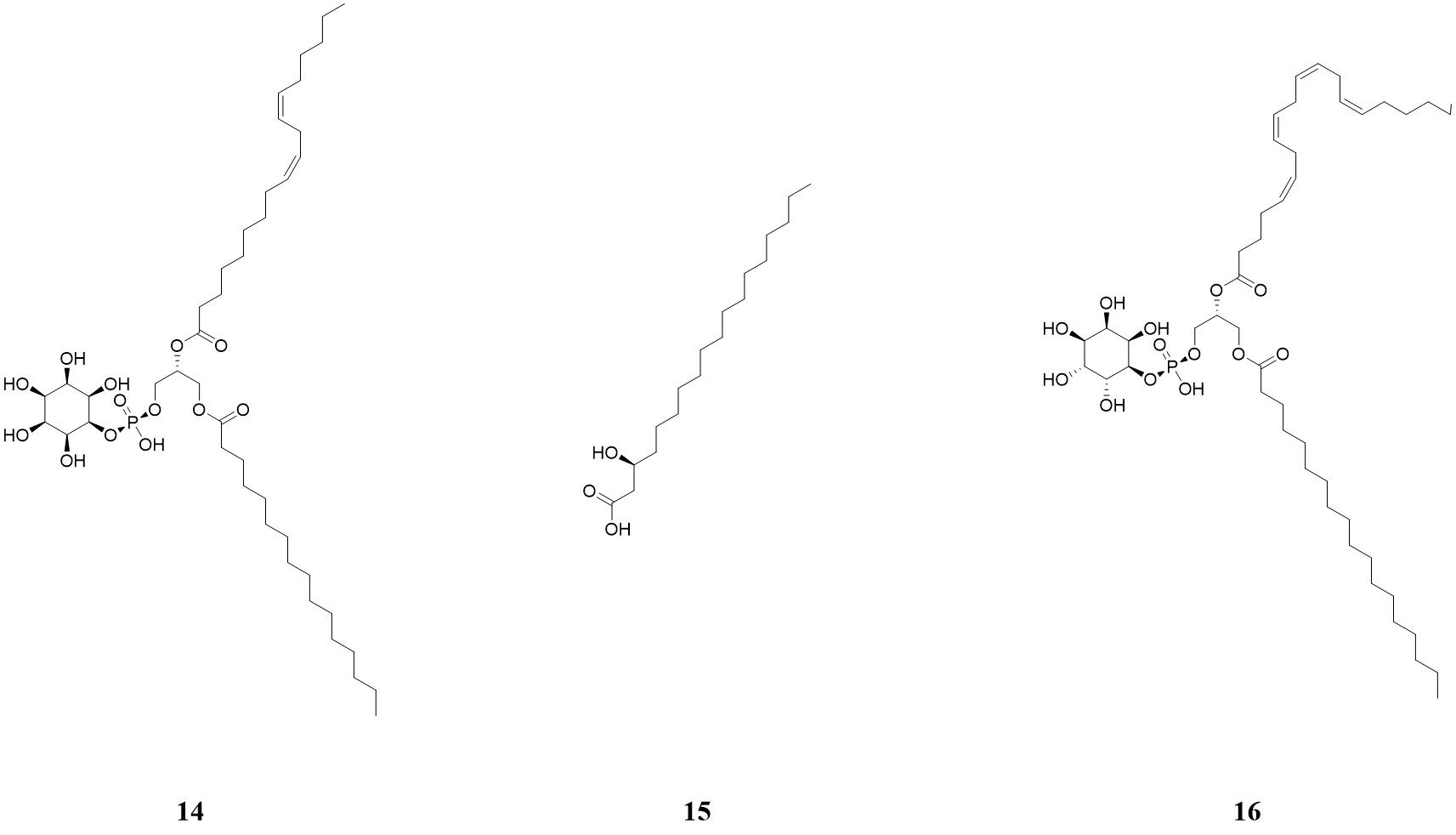


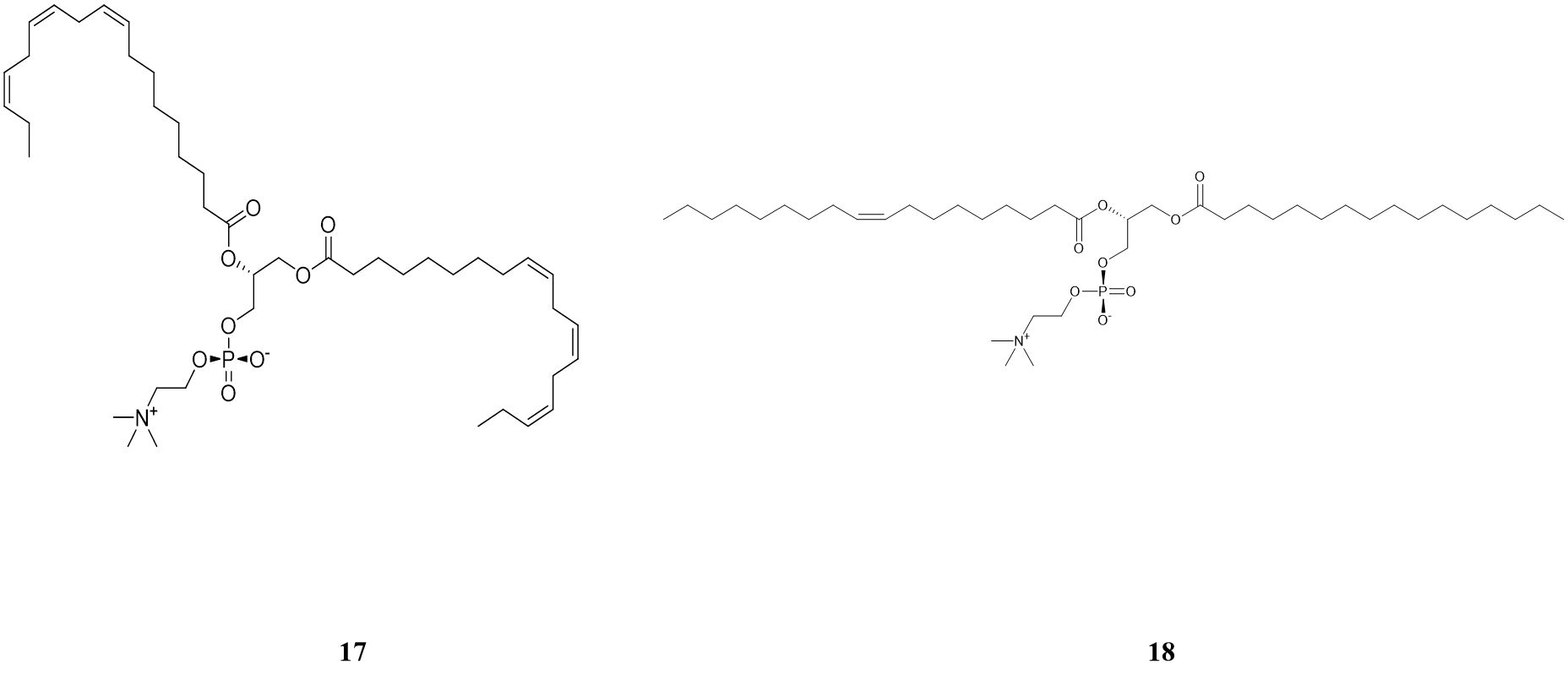


**Figure S4.** Mass Range obtained from the LC-MS of TVEAE. (A) Mass Range of TVEAE in positive ion modes. (B) Mass Range of TVEAE in negative ion modes.


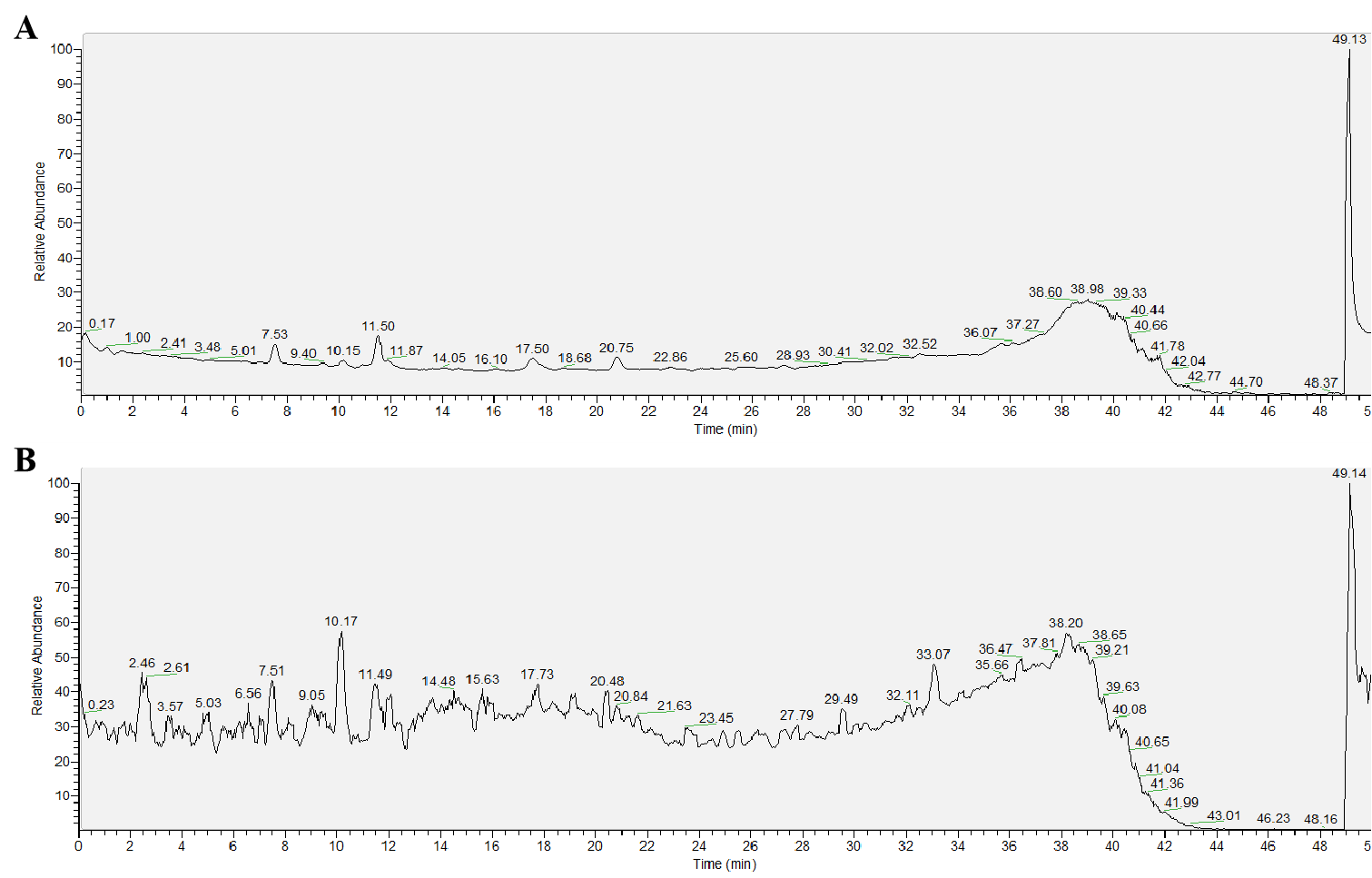


**Figure S5.** Diagram of Molecular Docking Results.


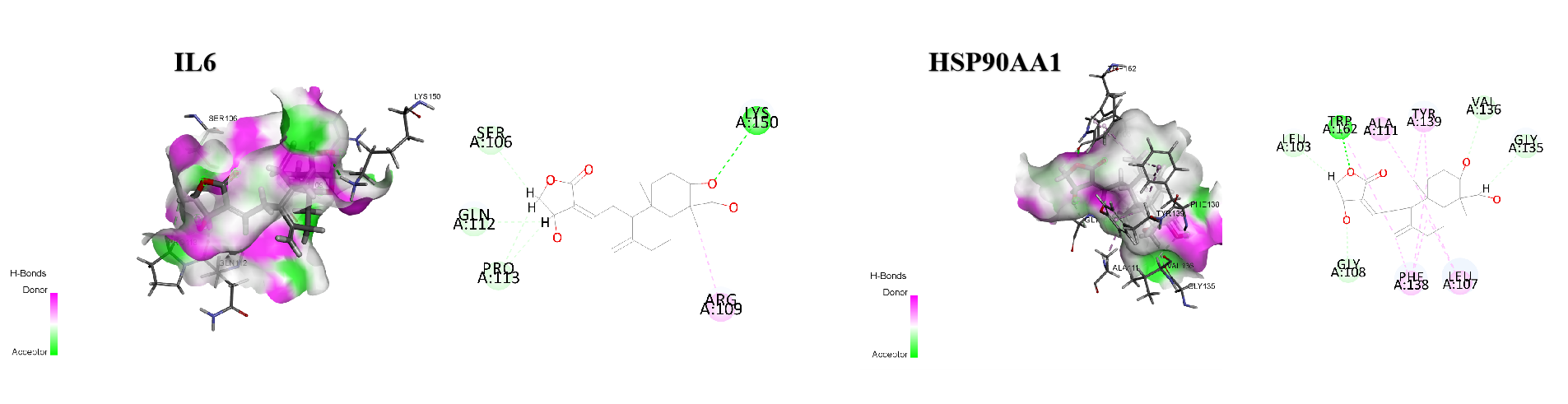


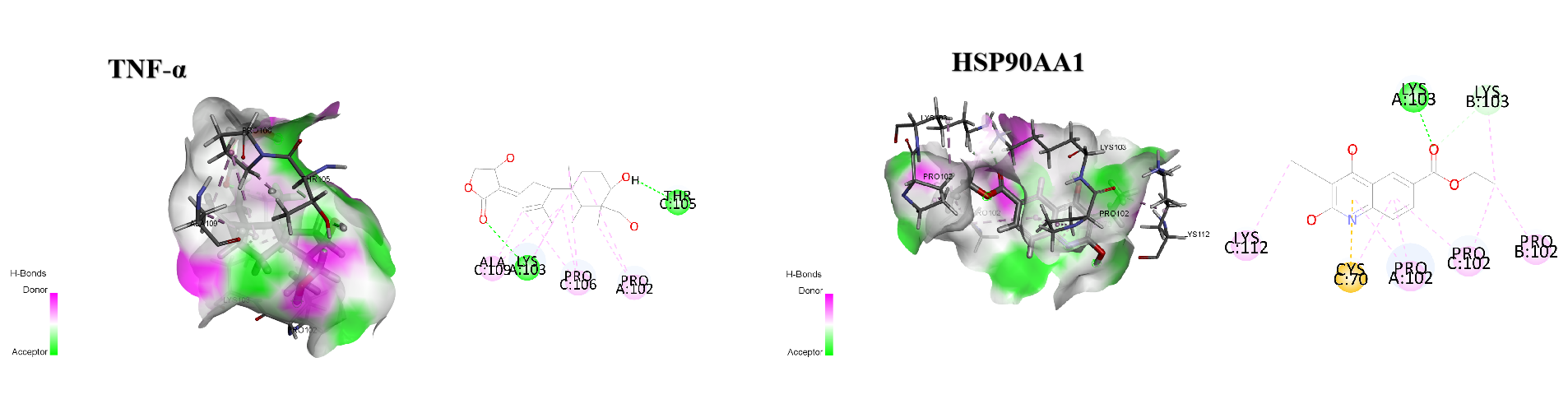


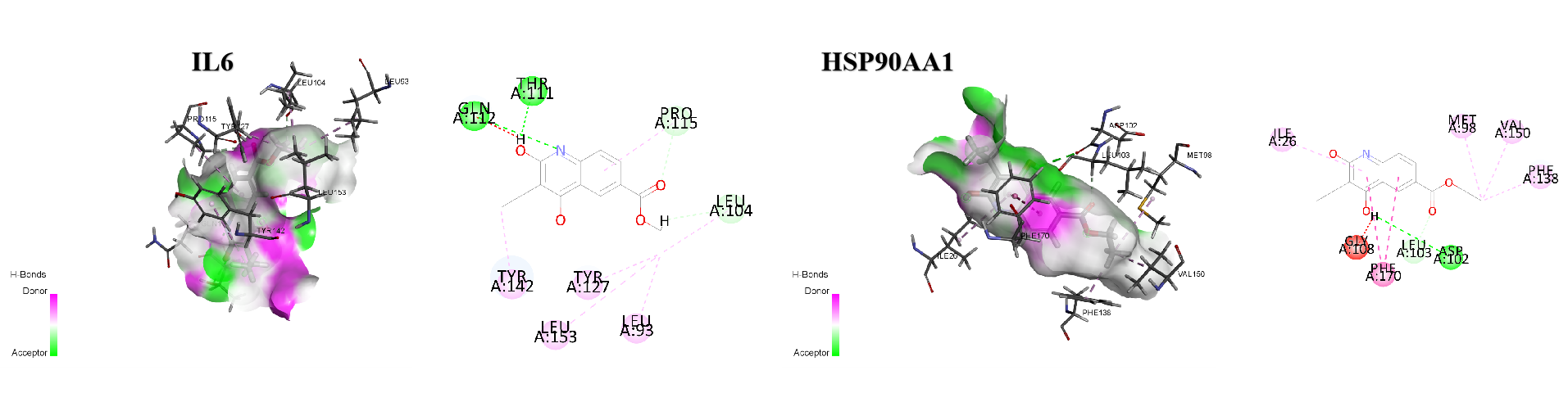


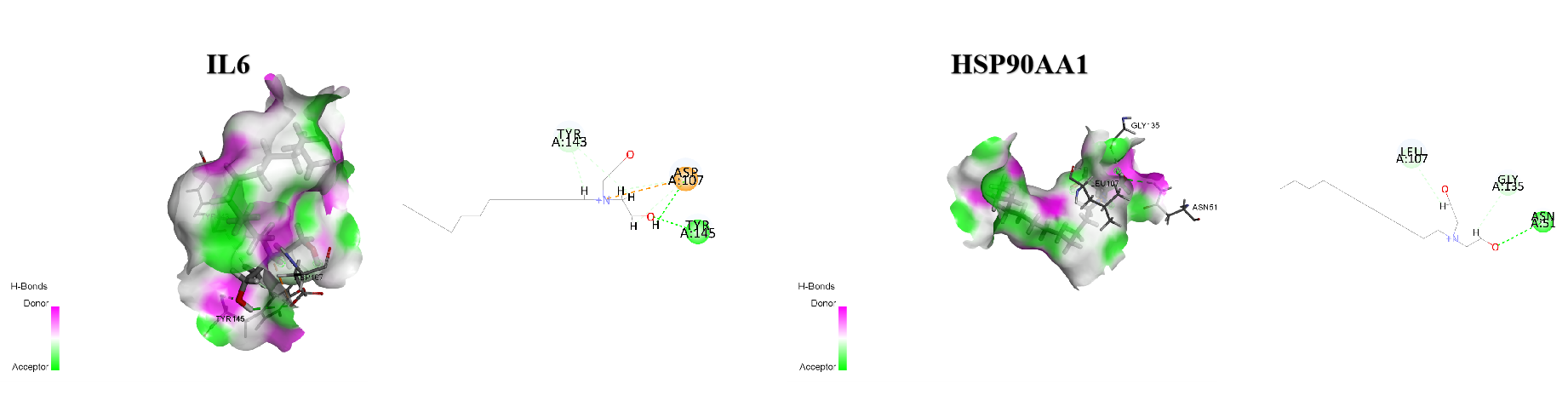


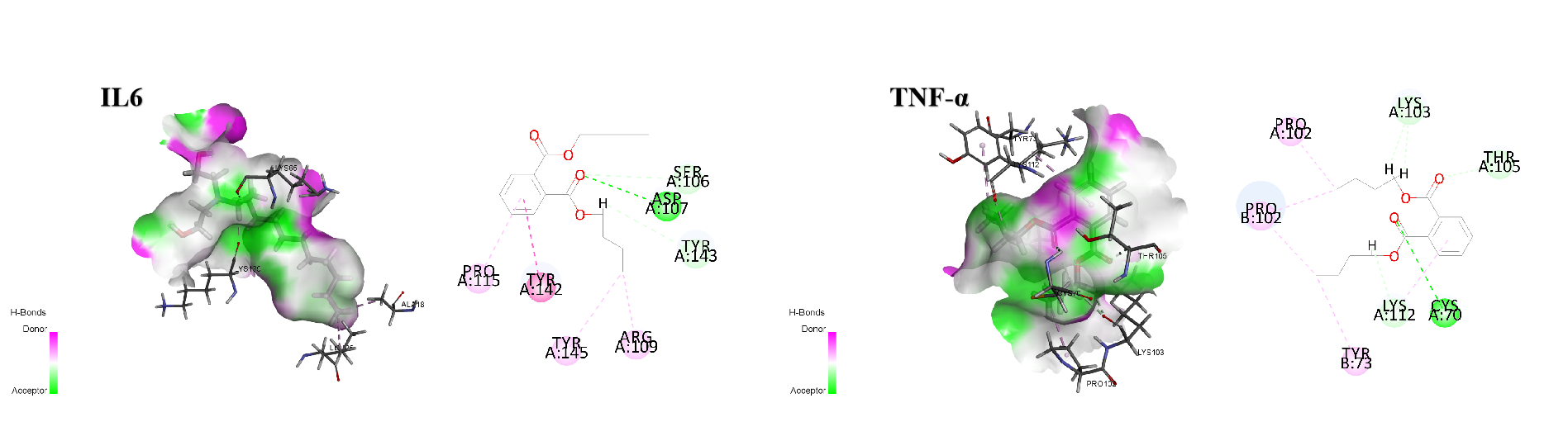


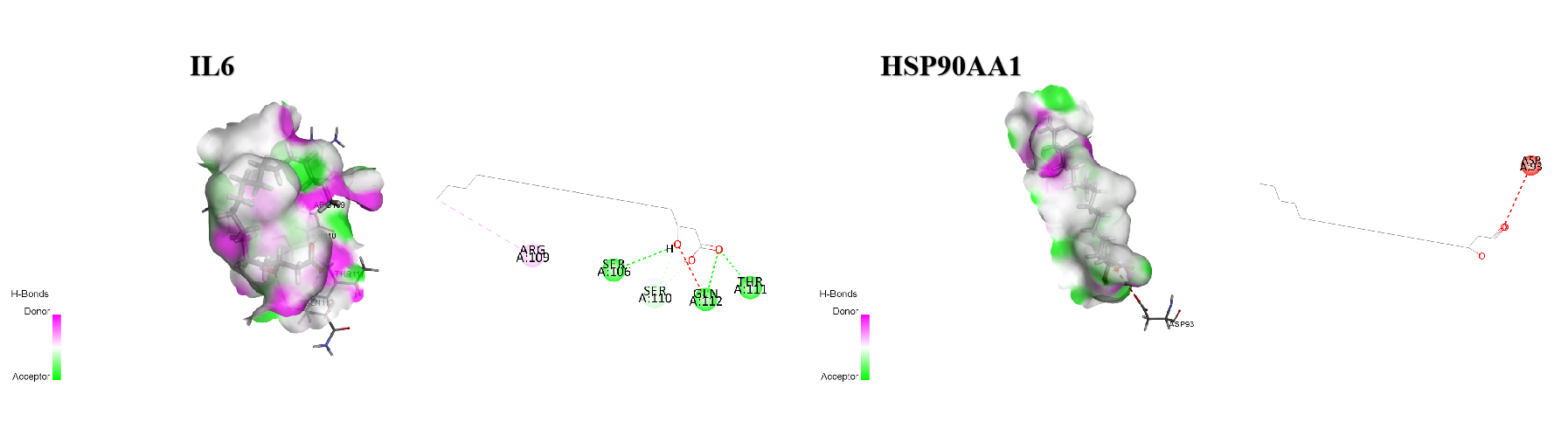


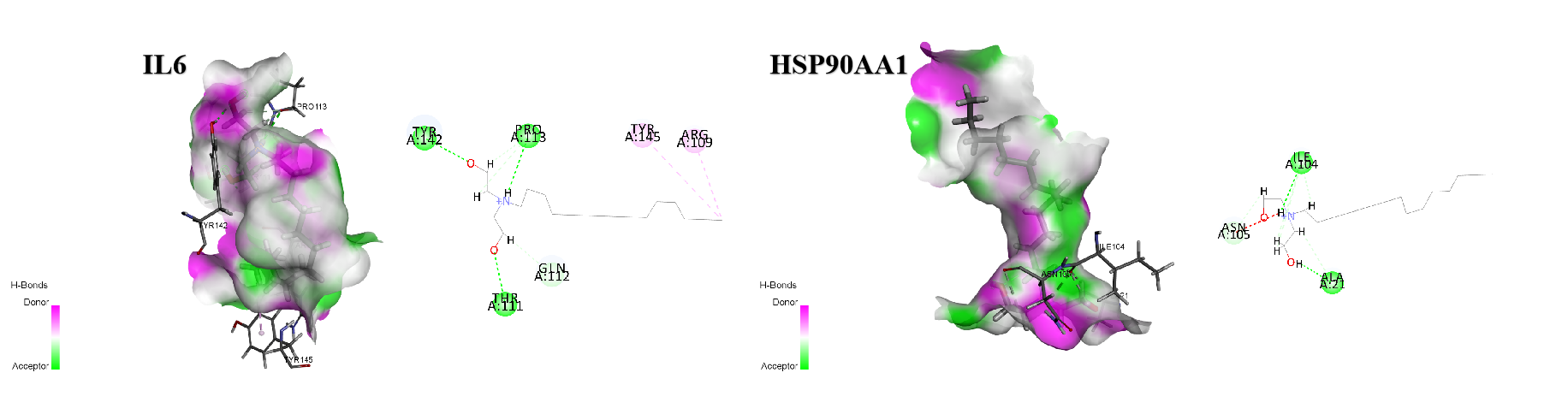


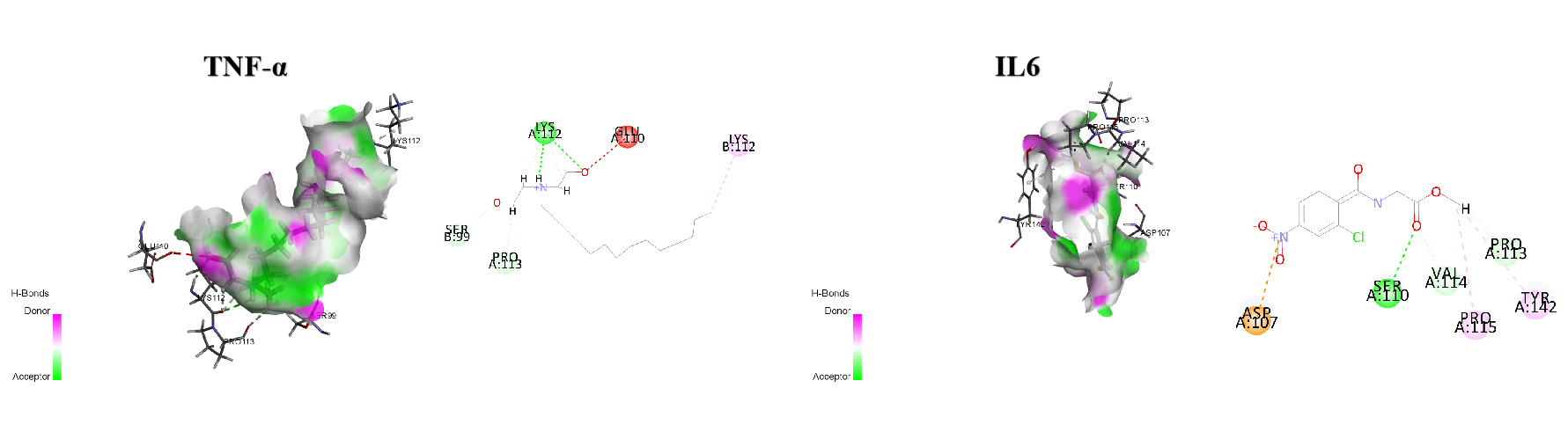


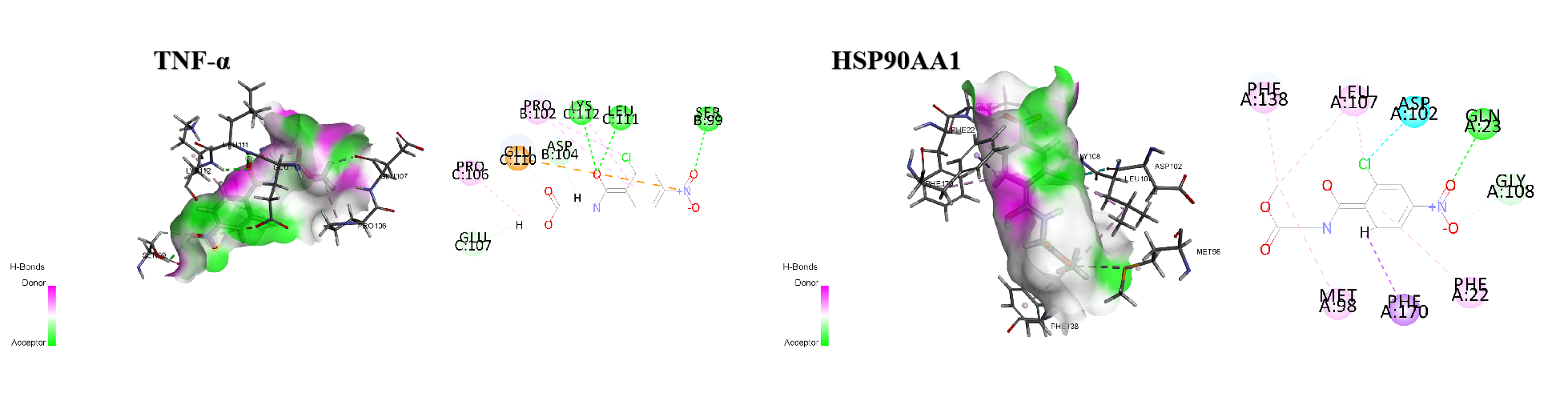


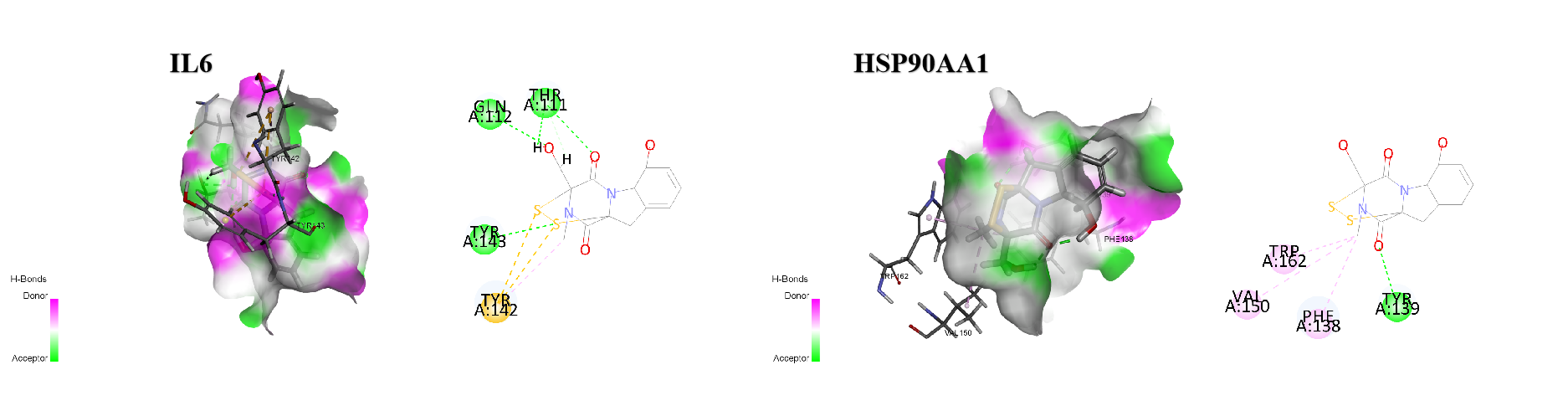


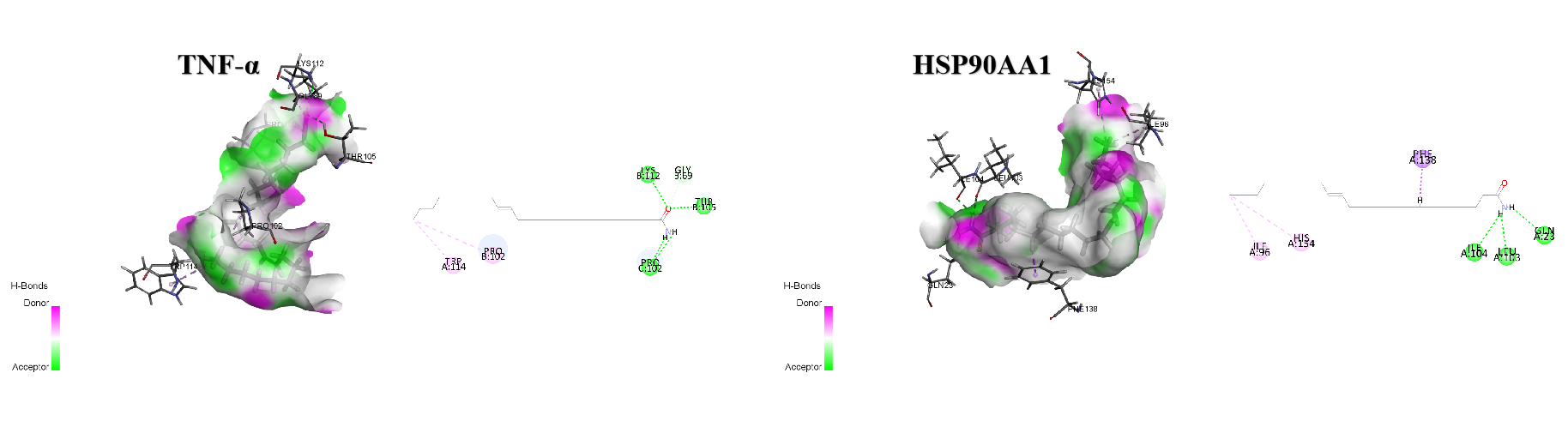


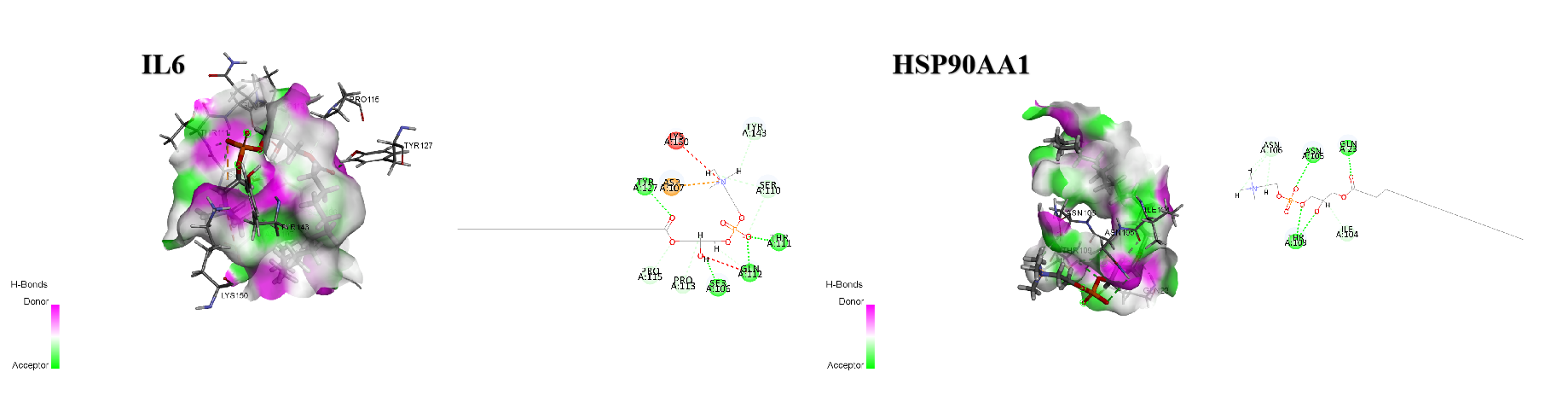


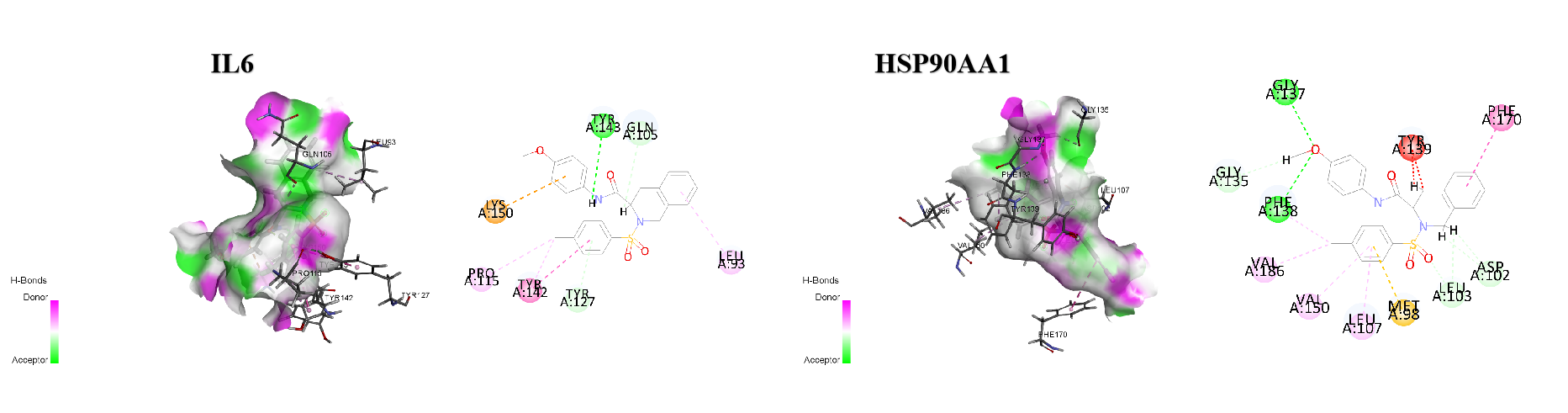


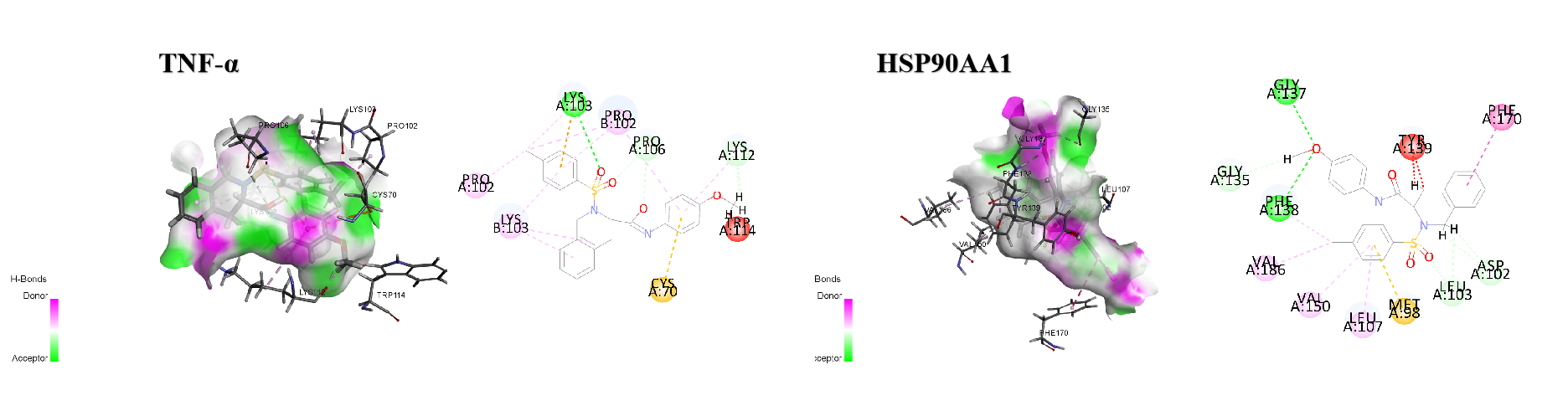


**Table S1**. Chemical constituents identified of TVEAE.

| **Compound** | ***t*_R_**  **(min)** | **Molecular**  **formula** | **Selected icon** | **Theoretical**  ***m/z*** | **Experimental**  ***m/z*** | **Error（ppm）** | ***MS/MS* fragmentation (m/z)** |
| --- | --- | --- | --- | --- | --- | --- | --- |
| **1** | 39.08 | C22H43NO | [M+H]^+^ | 337.60 | 337.3415 | -0.55797 | 254.2469、303.3046、321.3149 |
| **2** | 21.36 | C20H30O5 | [M+H]^+^ | 350.40 | 350.2166 | 0.03770 | / |
| **4** | 20.8 | C18H39NO2 | [M+H]^+^ | 301.5 | 301.30515 | -0.69710 | 88.0760、106.0864 |
| **7** | 24.72 | C24H50NO7P | [M+H]^+^ | 495.6 | 495.3393 | -0.27496 | 86.09680、104.10725、184.07313 |
| **8** | 11.25 | C13H14N2O4S2 | [M+H]^+^ | 326.4 | 326.11768 | 1.05960 | 201.0707、259.1023、309.1699 |
| **9** | 17.46 | C16H35NO2 | [M+H]^+^ | 273.45 | 273.27349 | -2.07842 | 88.07610、106.08652、256.26308 |
